# Evaluation of Diagnostic Potential of Epigenetically Deregulated MiRNAs in Epithelial Ovarian Cancer

**DOI:** 10.3389/fonc.2021.681872

**Published:** 2021-10-07

**Authors:** Vivek Kumar, Sameer Gupta, Amrita Chaurasia, Manisha Sachan

**Affiliations:** ^1^ Department of Biotechnology, Motilal Nehru National Institute of Technology, Allahabad, India; ^2^ Department of Surgical Oncology, King George Medical University, Lucknow, India; ^3^ Department of Gynaecology and Obstetrics, Motilal Nehru Medical College, Allahabad, India

**Keywords:** miRNA, EOC, expression biomarker, epigenetic regulation, EMT

## Abstract

**Background:**

Epithelial ovarian cancer (EOC) is one of the most lethal gynecological malignancies among women worldwide. Early diagnosis of EOC could help in ovarian cancer management. MicroRNAs, a class of small non-coding RNA molecules, are known to be involved in post-transcriptional regulation of ~60% of human genes. Aberrantly expressed miRNAs associated with disease progression are confined in lipid or lipoprotein and secreted as extracellular miRNA in body fluid such as plasma, serum, and urine. MiRNAs are stably present in the circulation and recently have gained an importance to serve as a minimally invasive biomarker for early detection of epithelial ovarian cancer.

**Methods:**

Genome-wide methylation pattern of six EOC and two normal ovarian tissue samples revealed differential methylation regions of miRNA gene promoter through MeDIP-NGS sequencing. Based on log2FC and *p*-value, three hypomethylated miRNAs (miR-205, miR-200c, and miR-141) known to have a potential role in ovarian cancer progression were selected for expression analysis through qRT-PCR. The expression of selected miRNAs was analyzed in 115 tissue (85 EOC, 30 normal) and 65 matched serum (51 EOC and 14 normal) samples.

**Results:**

All three miRNAs (miR-205, miR-200c, and miR-141) showed significantly higher expression in both tissue and serum cohorts when compared with normal controls (*p* < 0.0001). The receiver operating characteristic curve analysis of miR-205, miR-200c, and miR-141 has area under the curve (AUC) values of 87.6 (*p* < 0.0001), 78.2 (*p* < 0.0001), and 86.0 (*p* < 0.0001), respectively; in advance-stage serum samples, however, ROC has AUC values of 88.1 (*p* < 0.0001), 78.9 (*p* < 0.0001), and 86.7 (*p* < 0.0001), respectively, in early-stage serum samples. The combined diagnostic potential of the three miRNAs in advance-stage serum samples and early-stage serum samples has AUC values of 95.9 (95% CI: 0.925–1.012; sensitivity = 96.6% and specificity = 80.0%) and 98.1 (95% CI: 0.941–1.021; sensitivity = 90.5% and specificity = 100%), respectively.

**Conclusion:**

Our data correlate the epigenetic deregulation of the miRNA genes with their expression. In addition, the miRNA panel (miR-205 + miR-200c + miR-141) has a much higher AUC, sensitivity, and specificity to predict EOC at an early stage in both tissue and serum samples.

## 1 Introduction

According to the Cancer Statistics Report (2018), ovarian cancer (OC) was the seventh leading cause of cancer morbidity among American women. In 2018, more than 300,000 new cases were registered, with an incident rate of 11 per 100,000 women in Europe. A recent report of NCRP-2020 (National Cancer Registry Program, India) revealed that the incident rate of OC was around 9.5, making it the third most lethal gynecological malignancy among Indian women (1). Due to the asymptomatic progression of the disease and few screening options, ~70% of patients are presented at the advanced stage of ovarian cancer, leading to increased morbidity. Around 90% of OC cases are of epithelial subtypes. The most effective way to treat epithelial ovarian cancer (EOC) is surgery combined with chemotherapy; however, the final 5-year survival rate ranges between 35% and 50%, primarily due to EOC resistance to chemotherapy and recurrence of the disease (2). Existing diagnostic approaches, including cancer antigen 125 (CA125) level and physical examination methods (pelvic examination, imaging examination, ultrasound, transvaginal ultrasonography), are routinely used to diagnose EOC. However, early lesions were still complicated to be diagnosed, attributing to the low sensitivity of approximately 40% (3, 4). Because the ovaries are intraperitoneal organs, diagnosis of ovarian tumor is not possible without surgical resection (5). Therefore, the non-invasive and more precise biomarker test with superior diagnostic value should be developed, mainly for early diagnosis of EOC to reduce the morbidity and improve survival rate. Under this compelling scenario, recent advancements in global gene expression technology will pave a way to identify potential circulatory molecules like miRNA and cell-free DNA for cancer diagnosis.

MicroRNA is an evolutionarily conserved, non-coding, single-stranded RNA molecule of 22–25 nucleotides that negatively regulate target genes and modulate diverse biological processes, including development, differentiation, apoptosis, and proliferation of cells (6). Recent reports suggested that approximately 60% of human genes are regulated by miRNA at the translational level (7–10). A substantial amount of experimental evidence has shown that the aberrant expression patterns of miRNAs are associated with cancer progression and clinical features such as metastases, International Federation of Gynaecology and Obstetrics (FIGO) stage, and histology (11). MiRNA genes are located on the fragile location of the genome (cancer-associated genomic regions) and may be regulated by an epigenetic mechanism. In several cancer studies, aberrant expression of miRNA is reported to be coupled with extensive promoter hypermethylation and hypomethylation (12–15).

Similarly, several studies have demonstrated that deregulated miRNA expression is linked with ovarian cancer progression. The miRNA-20 family (miR-200c and miR-141) was found to be associated with epithelial–mesenchymal transition (EMT) and subsequently with ovarian cancer progression (16, 17). Similarly, oncogenic miR-205 was found to be involved in tumor formation *via* affecting cell proliferation and cell invasion (14). Despite these exciting researches, only limited data on the diagnostic potential of miRNA in EOC are available.

In the present study, we explored the genome-wide methylation pattern of miRNA genes in EOC samples [out of six EOC samples, two samples were from early stage (stage I–II) and four samples were from the advance stage (stage III–IV) and two normal samples] using methylated DNA immunoprecipitation (MeDIP)-NGS and correlated them with the differential expression pattern of selected candidate miRNA in EOC tissue and matched serum samples using quantitative real-time PCR (qRT-PCR). Moreover, we assessed the diagnostic potential of selected miRNAs for the prediction of EOC and the correlation of miRNA expression with clinical parameters. In addition, we also assessed the miRNA–target enrichment analysis and functional enrichment analysis of miRNA–targets.

## 2 Materials and Methods

### 2.1 Study Design, Patients, and Clinical Samples

Ovarian cancer tissue and matched preoperative serum samples were collected from the Department of Surgical Oncology, King’s George Medical University Lucknow, and histologically non-malignant tissue samples were collected from the Department of Gynecology and Obstetrics, Swaroop Rani Nehru Hospital, Prayagraj, with informed consent from the patients. All collected samples were processed and stored at −80°C. The study was approved by our Institutional Ethical Committee (IEC/2019-20/01). The clinicopathological data such as age, CA125 level, menopausal status, cancer histology, and FIGO stages were obtained from the record of the patient and pathological report from the source hospital.

**Figure 1:**
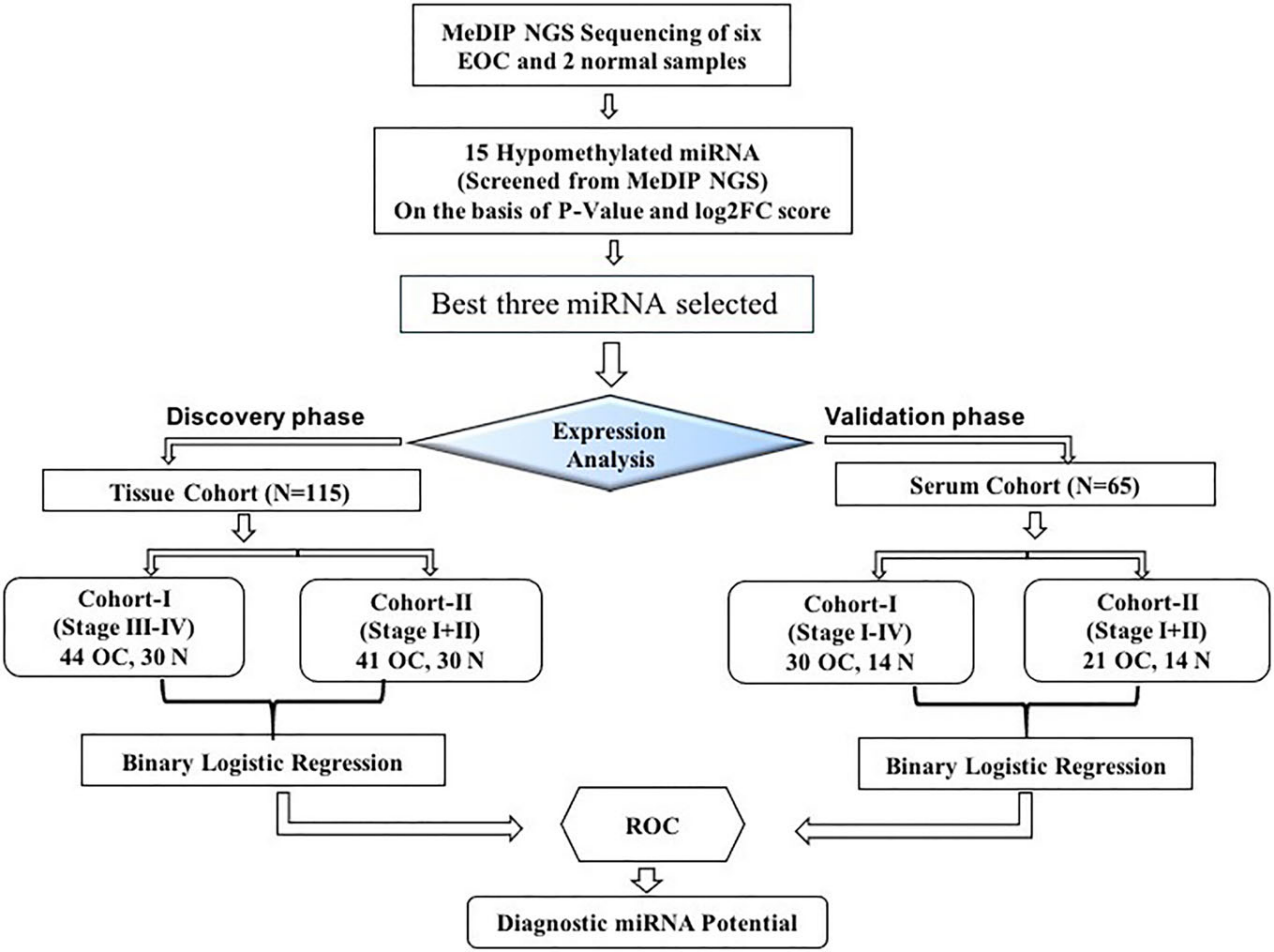
The layout of the study design. MeDIP, methylated DNA immunoprecipitation; EOC, epithelial ovarian cancer; ROC, receiver operating characteristic; N, normal.

This study was divided into two cohorts (tissue cohort and serum cohort); tissue cohorts were further divided into cohort-I [containing 44 advanced-stage (stage III–IV) EOC and 30 healthy control samples] and cohort-II (containing 41 early-stage EOC and 30 healthy control). Similarly, the serum cohort was divided into cohort-I [containing 30 advanced-stage (stage III-IV) EOC and 14 healthy samples] and cohort-II (including 21 early-stage EOC and 14 healthy samples) (**Figure 1**).

### 2.2 DNA Extraction

Around 20 mg of tissue samples were taken for the DNA extraction procedure. Tissue biopsy was washed in PBS buffer and homogenized in 2 ml SET buffer containing 0.3 M sucrose, 25 mM Tris (pH 8.0), and 5 mM EDTA (pH 8.0) at 4°C. Pellet obtained was lysed using 1 ml TEN buffer [50 mM Tris (pH 8.0), 2.5 mM EDTA (pH 8.0), and 100 mM NaCl] in the presence of 100 µl of 10% SDS and subjected to digestion with proteinase K (50 µg/ml) at 37°C overnight followed by phase separation using phenol:chloroform:isoamylalchohol (25:24:1). Furthermore, the supernatant was taken and precipitated with 1/30th volume of 3 M sodium acetate (pH 5.0) and two volumes of chilled absolute ethanol. After precipitation, the sample was subjected for centrifugation; the supernatant was discarded and the obtained pellet was air-dried and was further resuspended in 100–200 µl of TE buffer [10 mM Tris–HCl (pH 8.0) and 1mM EDTA (pH 8.0)]. Extracted DNA was further incubated at 37°C for 3–5 days. The quality and integrity of genomic DNA were assessed on 0.8% agarose gel at 120 V for 60 min or until the sample reached two-fourths of the gel. Furthermore, DNA was quantified using NanoDrop followed by a Qubit fluorometer and was stored at 4°C until further use.

### 2.3 MeDIP-Seq Library Preparation

QC (quality control)-passed gDNA was further used for the preparation of MeDIP sequencing libraries using Illumina TruSeq Nano DNA Library Prep Kit and MagMeDIP Kit (Diagenode, USA; Cat. No. C02010020) following the instruction of the manufacturer. Briefly, 1 μg of DNA from each sample was fragmented using Focused-Ultrasonicator (Covaris M220) to obtain the mean fragment distribution of 150 bp. Furthermore, fragments were processed for end-repair using iDeal Library End Repair/dA-Tailing Enzyme Mix (Diagenode, USA), followed by A-tailing and adaptor ligation. Next, the MagMeDIP kit was used for immunoprecipitation of the methylated DNA and enrichment by a short PCR cycle followed by AMPure XP bead purification. The purified PCR-enriched library of four samples (T55, T56, N66, and N65) was assessed on Agilent 4200 TapeStation system using sensitivity D1000 ScreenTape, while the remaining four samples (B10, T28, T21, and T65) were assessed on Agilent DNA HS chip system following the protocol of the manufacturer.

#### 2.3.1 Sequencing and Bioinformatics Analysis

After obtaining Qubit concentration for the libraries and mean peak sizes from the Agilent TapeStation profile, PE illumine libraries were loaded onto NextSeq 500 for cluster generation and sequencing generating around 56 to 80 million reads per sample. The reads were processed to obtain high-quality clean reads using the Trimmomatic tool by applying the following filters: remove adapter sequences, ambiguous reads (reads with unknown nucleotides “N” larger than 5%), and low-quality sequences [reads with more than 10% quality threshold (QV) <20 Phred score]. After removing all ambiguous reads and adapter, high-quality reads were retained for all samples, respectively, and further paired-end reads were used for referenced-based read mapping. Using BWA-Mem tools with default parameters, high-quality reads were mapped to the Hg19 genome. Furthermore, the aligned files were processed by samtools (v1.6) to convert the alignment output into bam file with the filter to include only mapping with properly paired read pair tag, and mapping quality of 1 and above were retained for further analysis. In addition, in cases where multiple reads pair with identical coordinates, the pair with the highest mapping quality was considered, and duplicated reads were removed using samtools (v1.6) package.

#### 2.3.2 Methylated Genome Regions and Differential Analysis

MeDIP enables the rapid identification of genomic regions containing methylated cytosines. MeDIP, in combination with high-throughput sequencing (HTS) techniques, is a useful method for identifying methylated CpG-rich sequences. After post-alignment processing, bam files were directly screened for the methylated region using diffReps (v1.55.6), by the following filter: window size 1,000 bp, step size: 100 bp, statistical testing: G-test, and *p*-value <0.05. The reason behind using partially overlapping windows is to increase the resolution of differential site detection. The diffReps interprets the up/downregulation based on the normalized read count and performs the G-test on the log fold change values to calculate the *p*-value and *q*-value.

### 2.4 MiRNA–Target Prediction and KEGG Pathway Enrichment Analysis

Six online tools (miRDB, Tools4miRs, TargetScanHuman7.2, miRWalk2.0, miRanda, RNAhybrid) were used to identify putative target genes for three candidate miRNAs (18–21). Relation between identified target genes and candidate miRNA was manually curated and experimentally validated in the database. Only overlapped target genes from six online tools were selected for the study. Furthermore, miRNA–disease association network enrichment analysis was performed to see the association of target miRNA with the disease. To build a disease–miRNA enrichment network, the online tool miRNet v.20 was used, and the parameter was only for ovarian cancer or ovarian neoplasm (22). Furthermore, miRNA–target genes regulatory network analysis was performed by uploading identified putative target genes in online tools (mirnet.ca). The created networks reveal functional relationships between miRNAs and genes based on known associations in the database (23). In addition, to exploit the functions of predicted target genes, we performed Gene Ontology (GO) using GO stat package, while the Kyoto Encyclopedia of Genes and Genomes (KEGG) pathway enrichment analysis was performed using DAVID (https://david.ncifcrf.gov/), with default parameter (enrichment score and *p <*0.05 as a cutoff value for the selection of enriched function) (24).

### 2.5 MiRNA Extraction From Tissue and Serum Samples

Total miRNA was isolated from 85 EOC and 30 healthy tissue samples using miRNeasy Mini Kit (Cat. No. 217004, Qiagen) following the protocol of the manufacturer. Similarly, serum miRNA was isolated from 65 serum samples using miRNeasy Serum/Plasma Kit (Cat. No. 217184; Qiagen). For normalization, 3.5 µl of Spike-In Control (1.6 × 10^8^ copies) (*Caenorhabditis elegans* miRNA cel-miR-39) was added to each sample. Isolated miRNAs from tissue and serum samples were quantified using a microspectrophotometer (DeNovix, USA). The yield of miRNA for individual tissue samples ranges from 0.4 to 2.5 µg, while for serum miRNA, it was 0.2 to 0.4 µg.

### 2.6 MiRNA Quantification and Normalization

The expression level of selected miRNA in tissue and serum was quantified by qRT-PCR. Total 1.0 µg miRNA from tissue and total miRNA from serum samples were polyadenylated using poly(A) polymerase and reverse transcribed using miScript^®^ II RT Kit (Cat. No. 218160, Qiagen) following the instruction of the manufacturer. Furthermore, complementary DNA (cDNA) was diluted to bring the final concentration to 8 ng/µl for subsequent real-time qPCR reaction and stored at −80°C. The quantitative expression analysis of selected miRNA was performed according to SYBR^®^ Green PCR Kit (Cat. No. 218073, Qiagen) with target miRNA primer (miScript Primer Assay, Cat. No. 218300, Qiagen, India) using StepOne™ Plus Real-Time PCR machine (Applied Biosystems). All qRT-PCR reaction was performed in triplicate as per the protocol of the manufacturer with the following cycling conditions: initial activation step for 15 min at 95°C, three-step cycling includes denaturation at 94°C for 15 s, annealing at 55°C for 30 s, and extension at 70°C for 30 s, for 40 cycles. For normalization, miRNA-191 was taken as an endogenous control for tissue miRNAs, while cel-miR-39 (Spike-In Control, Qiagen) and miRNA-191 were combined to normalize serum miRNA. The equation used for the normalization of serum miRNA was ΔCt (Ct_miRNA_ − 0.5 * (Ct_cel-miR-39_ + Ct_miR-191_) (25).

### 2.7 Statistical Analysis

The relative expression was calculated using the LIVAK method (2^−ΔΔCT^). The data were presented as mean and standard deviation and the categorical variables as count or percentage. For the assessment of differences in two groups of continuous variables, the Mann–Whitney *U* test was performed. One-way ANOVA was performed for comparison between more than two groups of a continuous variable. To evaluate the correlation between the two groups, Spearman’s rank-order correlations were used. Univariate logistic regression analysis of individual miRNA and binary logistic regression analysis were performed, enabling the estimation of predicted probabilities of the miRNA panel, which was further used to produce area under the curve (AUC), sensitivity, and specificity. Sensitivity and specificity were given equal weightage to evaluate the optimal cutoff value, accuracy, and AUC.

All statistical test was conducted in SPSS^®^ (Version 26, SPSS Inc., Chicago, USA), and the graph was plotted in GraphPad Prism (Version 9.0). All statistical analysis was two-sided, and *p*-value <0.05 was considered statistically significant.

## 3 Results

### 3.1 Clinical Characteristics of Patients

We recruited 115 tissue samples (85 EOC and 30 healthy) and 65 preoperative matched serum samples (51 EOC and 14 healthy) and further divided them into four histological groups based on the pathological report; the cases included 51 serous (60.0%), 15 mucinous (16.4%), 10 clear cell (11.7%), and 10 endometrioid (12.9%). Samples were further categorized according to FIGO stages; the cases included 25 stage I (29.4%), 16 stage II (18.8%), and 44 stage III–IV (51.7%). Similarly, serum samples were subdivided on the basis of histology; the cases included are 28 serous (54.9%), 11 mucinous (22.0%), 6 clear cell (12.0%), and 6 endometroid (12.0%), respectively. Detailed clinical characteristics of the tissue cohort and serum cohort are summarized in **Table 1** (A = tissue, B = serum).

**Table 1:** Clinical characteristics of samples recruited for this study.

Clinical characteristics of patient enrolled in this study											
(A) Tissue cohort (*n* = 115)						(B) Serum cohort (*n* = 65)					
Variables	Case (*n* = 85)	Control (*n* = 30)	Relative expression of miR-205 (2^−ΔΔCT^)	Relative expression of miR-200c (2^−ΔΔCT^)	Relative expression of miR-141 (2^−ΔΔCT^)	Variables	Case (*n* = 51)	Control (*n* = 14)	Relative expression of miR-205 (2^−ΔΔCT^)	Relative expression of miR-200c (2^−ΔΔCT^)	Relative expression of miR-141 (2^−ΔΔCT^)
Age, *n* (%)						Age, *n* (%)					
<45	26 (30.5)	16 (53.3)	4.49 ± 1.88	5.12 ± 2.94	5.64 ± 3.15	<45	12 (24.0)	–	7.50 ± 4.82	6.02 ± 4.40	5.74 ± 4.44
≥45	59 (69.4)	14 (46.6)	4.24 ± 2.84	4.22 ± 2.56	5.62 ± 3.66	≥45	38 (76.0)	–	4.69 ± 3.58	3.77 ± 3.05	4.64 ± 3.18
*p*-value			ns	ns	ns				ns	ns	ns
Histological type, *n* (%)						Histological type, *n* (%)					
Mucinous	15 (16.4)	–	6.08 ± 2.76	4.53 ± 2.85	7.08 ± 4.01	Mucinous	11 (22.0)	–	4.47 ± 3.89	2.69 ± 2.06	4.01 ± 2.64
Serous	51 (60.0)	–	3.76 ± 2.14	4.20 ± 2.59	4.84 ± 3.26	Serous	28 (54.9)	–	5.31 ± 4.54	4.80 ± 3.87	5.28 ± 3.50
Clear cell	10 (11.7)	–	3.47 ± 1.33	4.01 ± 2.41	6.48 ± 3.25	Clear cell	6 (12.0)	–	6.18 ± 3.79	5.81 ± 4.00	5.54 ± 5.24
Endometrioid	11 (12.9)	–	5.44 ± 3.77	6.23 ± 2.90	5.05 ± 3.24	Endometrioid	6 (12.0)	–	5.90 ± 2.84	3.56 ± 2.84	4.35 ± 3.46
Other	–	–	–	–	–	Other	–	–	–	–	–
*p*-value			< 0.005	<0.005	<0.05				ns	ns	ns
Distant metastases, *n* (%)						Distant metastases, *n* (%)					
Absent	41 (48.2)	–	2.18 ± 1.99	2.29 ± 1.96	2.03 ± 3.36	Absent	21 (41.1)	–	3.73 ± 2.91	2.46 ± 1.19	3.09 ± 1.82
Present	44 (51.7)	–	6.69 ± 2.75	6.73 ± 2.09	6.47 ± 3.56	Present	30 (58.8)	–	6.55 ± 4.25	5.20 ± 4.19	6.11 ± 3.85
*p*-value			<0.0001	<0.0001	<0.0001				<0.001	<0.001	<0.001
FIGO stage, *n* (%)						FIGO stages, *n* (%)					
I–II	41 (48.2)	–	3.57 ± 2.06	3.91 ± 2.43	4.48 ± 3.04	I–II	21 (41.1)	–	3.14 ± 2.61	2.58 ± 2.13	2.94 ± 2.08
III–IV	44 (51.7)	–	4.97 ± 2.86	5.02 ± 2.83	6.27 ± 3.61	III–IV	30 (58.8)	–	6.83 ± 4.19	5.46 ± 3.80	6.21 ± 3.67
*p*-value			<0.05	<0.005	<0.005				<0.05	<0.005	<0.005
Menopause status, *n* (%)						Menopause status, *n* (%)		–			
Yes	51 (60.0)		4.58 ± 2.76	4.47 ± 2.72	5.77 ± 3.50	Yes	38 (76.0)		5.26 ± 3.84	4.06 ± 3.22	5.23 ± 3.57
No	26 (40.0)		3.94 ± 2.54	4.54 ± 2.70	5.41 ± 3.40	No	12 (24.0)		5.66 ± 4.73	5.01 ± 4.31	3.99 ± 3.25
*p*-value			ns	ns	ns				ns	ns	ns
Serum CA125 (U/ml)	173 ± 329.1	33.8 ± 61.4				Serum CA125 (U/ml)	172 ± 352.4	–			

Categorical variables are presented as percentage; continuous variables are presented as mean ± SD (standard deviation); statistically significant differences were determined by the Mann–Whitney U test and one-way ANOVA test.

FIGO, International Federation of Gynaecology and Obstetrics.

### 3.2 MeDIP-Seq Analysis of Six EOC and Two Normal Samples

**Figure 2:**
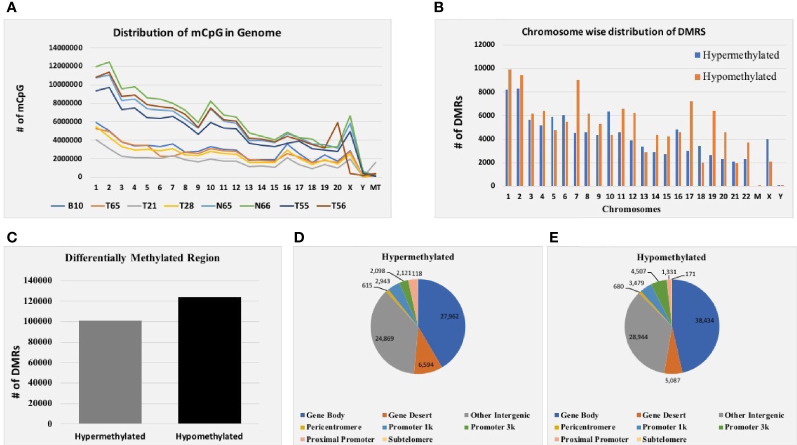
MeDIP-seq results. **(A)** Distribution of methylated CpG in each chromosome of eight EOC tissue samples. **(B)** Total distribution of hypo- and hypermethylated differentially methylated regions (DMRs) at each chromosome of eight tissue samples. **(C)** Total hyper- and hypomethylated DMRs obtained from eight EOC tissue samples. **(D)** Distribution of hypermethylated DMRs in different genomic regions. **(E)** Distribution of hypomethylated DMRs in different genomic regions of eight EOC tissue samples.

Genomic DNA from six EOC and two normal tissue samples were isolated, and the DNA was analyzed for integrity and concentration. Furthermore, MeDIP-seq was conducted using the Illumina NextSeq 500 platform, which provides precise accuracy and high-quality output. The mean average size of the library profile of each sample was around 300 bp. After the removal of low-quality sequencing data, ~9.7 Gb of average bases were sequenced per sample. Furthermore, in the bioinformatics analysis of MeDIP-seq data, we obtained ~61.0–80.0 million clean reads per sample. Next, we mapped all the clean reads against the reference genome. The mapping percentage ranges between 80.30% and 92.67% for each sequenced sample (**Supplementary Table 1**). In addition, genome-wide distribution of methylated CpG is depicted in **Figure 2A**.

#### 3.2.1 Differentially Methylated Regions in Six EOC and Two Normal Samples

Furthermore, mapped reads were curated to identify differentially methylated regions (DMRs) in EOC and normal samples. A sum of 224,929 DMRs (*p* < 0.05; FC ≥ 2) was identified using diffReps software along with G-test. Out of them, 101,218 (45%) were hypermethylated, and 123,711 (55%) were hypomethylated (**Figure 2C**). Next, we analyzed the chromosome-wise distribution of hyper- and hypomethylated DMRs (**Figure 2B**). In addition, we also assessed the genomic distribution of DMR in different regions of the chromosome, and we found that the majority of hypermethylated DMRs were enriched in the gene body followed by other intergenic regions of the genome. Similarly, most hypomethylated DMRs were enriched in the gene body, followed by other intergenic regions of the genome (**Figures 2D, E**). Furthermore, hypermethylated genomic intervals were annotated using the tool region analysis (v1.0). Gene information obtained from Ensembl was used while performing annotation.

#### 3.2.2 Identification of Best-Hypomethylated miRNA

The location of the miRNA putative promoter region was identified using three different tools (TransmiR v2.0, microTSS, and miRstart). Furthermore, overlapping predicted miRNA promoter regions were selected and manually screened in identified DMRs for hypomethylated and hypermethylated status. The top 15 hypomethylated miRNA DMRs were screened based on log2FC and *p*-value, and among them, three candidate miRNAs were selected on the basis of the best *p*-value and log2FC value for further analysis (**Supplementary Table 2**).

### 3.3 Association of MiRNA Expression and Hypomethylation of MiRNA Gene

We performed expression analysis of candidate miRNA in eight samples (used for MeDIP-seq) to establish the association of miRNA gene methylation and its downstream expression. The mean relative expression of miR-205, miR-200c, and miR-141 was significantly elevated in six EOC samples compared with the normal samples with fold change of 3.85 (*p* < 0.05), 4.95 (*p* < 0.05), and 4.69 (*p* < 0.05), respectively (**Supplementary Figure 1**). On the basis of methylation status of miRNA in these eight tested samples and their downstream expression analysis, we are able to conclude that the expression of candidate miRNA is dependent upon epigenetic changes that occurred on their gene. To further validate the expression level of these miRNAs, we performed expression analysis of the remaining 107 samples.

### 3.4 Expression Analysis of Candidate MiRNAs in Tissue Cohorts

**Figure 3:**
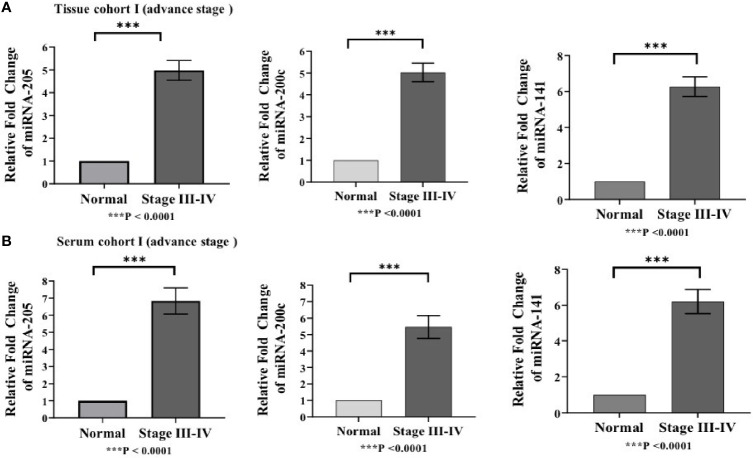
Represents upregulation of miRNAs in epithelial ovarian cancer in cohort I of tissue and serum both. **(A)** In tissue cohort-I {n=74 (EOC=44, Normal=30)}, expression levels of miR-205, -200c, and miR-141 were increased in the cancer group by 4.98(P < 0.0001), 5.03 (P < 0.0001), and 6.27 (P <0.0001) fold respectively when compared with the control group. **(B)** In serum cohort-I{n=45 (EOC=30, Normal=15)}, the fold change of miR-205, miR-200c and miR-141 was 6.83 (P <0.0001); 5.46(P<0.0001) and 6.21 (P<0.0001) respectively. Statistically significant differences were determined by the Mann Whitney U-tests. Data represent mean ± standard error on the mean (SEM). ***P < 0.0001.

**Figure 4:**
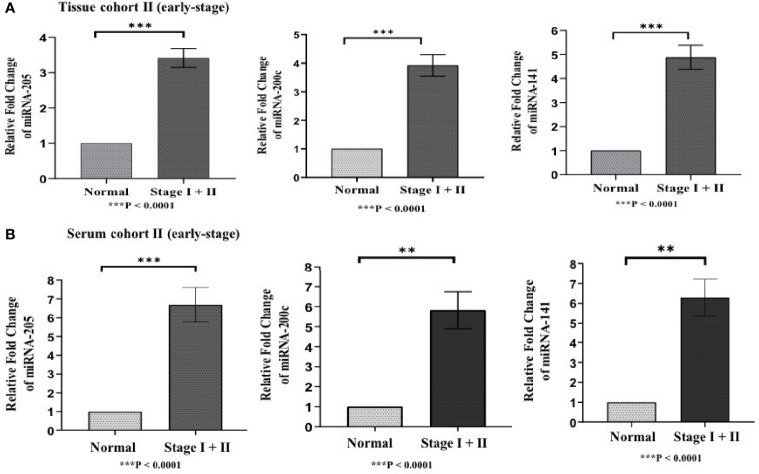
Represents upregulation of miRNAs in epithelial ovarian cancer in cohort II of tissue and serum both. **(A)** In tissue cohort-II {n=71 (EOC=41, Normal=30)}, expression levels of miR-205, -200c, and miR-141 were significantly increased in the cancer group compared with the control group by 3.63 (P < 0.0001), 3.93 (P<0.0001) , 4.94 (P<0.0001) fold respectively. **(B)** In serum cohort-II {n=35 (EOC=21, Normal=14)}, the fold change of miR-205, miR-200c and miR-141 was 6.70 (P<0.0001), 5.82 (P = 0.0063), and 6.29 (p < 0.001) respectively. Statistically significant differences were determined by the Mann Whitney U-tests. Data represent mean ± standard error on the mean (SEM). **P < 0.01; ***P < 0.0001.

Differences in the expression level of selected miRNAs (miR-205, miR-200c, and miR-141) were quantified using qRT-PCR in both tissue cohorts and compared with the healthy samples. In tissue cohort-I, relative expression of miR-205, miR-200c, and miR-141 was found significantly elevated in EOC samples as compared to normal control with the respective fold change 4.98 (*p* < 0.0001), 5.03 (*p* < 0.0001), and 6.27 (*p* < 0.0001) (**Figure 3A**), respectively. Similarly, in cohort-II, relative expression of miR-205, miR-200c, and miR-141 exhibited higher expression in EOC when compared to control samples with a fold change of 3.63 (*p* < 0.0001), 3.93 (*p* < 0.0001), and 4.94 (*p* < 0.0001), respectively (**Figure 4A**).

### 3.5 Expression Analysis of Candidate MiRNAs in Serum Cohorts

To validate the consistency of overexpression, the relative expression of these miRNAs was analyzed in 65 serum samples. In serum cohort-I, miR-205, miR-200c, and miR-141 were significantly elevated in EOC samples as compared with those in healthy samples, and their respective fold change was 6.83 (*p* < 0.0001), 5.46 (*p* < 0.0001), and 6.21 (*p* < 0.0001) (**Figure 3B**). Similarly, in serum cohort-II, miR-205, miR-200c, and miR-141 were significantly elevated in the EOC sample with the respective fold change of 6.70 (*p* < 0.0001), 5.82 (*p* < 0.0001), and 6.29 (*p* < 0.001) (**Figure 4B**).

### 3.6 Correlation Between Candidate MiRNA Expression and Clinical Parameters

The association between the relative expression level of miRNAs and clinical parameters were evaluated for both tissue (85 EOC patients) and serum (50 EOC patients) cohorts. While comparing FIGO stages, the expression level of miR-205, miR-200c, and miR-141 was significantly increased in combined stage III–IV of EOC patients in comparison with that in stage I and stage II (*p*-value = <0.05, <0.005, and <0.005, respectively) in tissue cohorts (**Supplementary Figure 2A**). Based on clinicopathological features, patients were divided into four subgroups (namely, serous, mucinous, clear cell, and endometrioid). The mean expression level of miR-205 was significantly elevated in mucinous subtypes (*p* < 0.005), while miR-200c was significantly elevated in endometrioid subtypes (*p* < 0.005). Similarly, the expression level of miR-141 was significantly elevated in the serous subtype (*p* < 0.05) compared with that in other histotypes (**Supplementary Figure 3A**). Similarly, the mean expression level of the three candidate miRNAs were considerably elevated in metastatic patients as compared with that in non-metastatic patients (*p*-value = <0.0001, <0.0001, and <0.0001, respectively) (**Supplementary Figure 4A**).

In addition, the expression level of all three miRNAs was considerably increased in patients with combined FIGO stage III–IV in comparison with combined FIGO stage I–II (*p* < 0.05, *p* < 0.005, and *p* < 0.005, respectively) in the serum cohort (**Supplementary Figure 2B**). However, the elevated expression of miR-205, miR-200c, and miR-141 could not discriminate any of the histological subtypes (**Supplementary Figure 3B**). Like EOC tissue samples, all tested miRNAs were significantly elevated in serum samples of metastatic patients (*p*-value < 0.001, <0.001, and <0.001, respectively) (**Supplementary Figure 4B**).

In addition, we analyzed the correlation of expression of the three miRNAs with the age, menopause status, CA125 level, and distant metastasis of the patient. In tissue cohort-I and cohort-II, age were not correlated with any tested miRNA. In tissue cohort-I, miR-205, miR-200c, and miR-141 were positively correlated to CA125 level (*r* = 0.380, *p* = 0.001; *r* = 0.458, *p* = 0.0001; *r* = 0.428, *p* = 0.0001, respectively) and distant metastases (*r* = 0.353, *p* = 0.002; *r* = 0.417, *p* = 0.0001; *r* = 0.333, *p* = 0.004, respectively). In tissue cohort-II, miR-200c was positively correlated with menopausal status, while miR-141 was positively correlated with CA125 level. Since tissue cohort-II only consist of early samples, the correlation was nullified during analysis (**Supplementary Table 3**).

Similarly, in serum cohorts, miR-205 and miR-141 were positively correlated with age (*r* = 0.477, *p* = 0.001; *r* = 0.330, *p* = 0.031, respectively). On the other hand, the expression level of miR-205, miR-200c, and miR-141 was positively correlated with CA125 (*r* = 0.455, *p* = 0.002; *r* = 0.570, *p* = 0.0001; *r* = 0.471, *p* = 0.001, respectively), menopausal status (*r* = 0.617, *p* = 0.0001; *r* = 0.463, *p* = 0.002; *r* = 0.591, *p* = 0.0001, respectively), and distant metastases (*r* = 0.462, *p* = 0.002; *r* = 0.357, *p* = 0.017; *r* = 0.484, *p* = 0.001, respectively) in serum cohort-I. In addition, miR-205, miR-200c, and miR-141 were positively correlated with CA125 level (*r* = 0.483, *p* = 0.003; *r* = 0.378, *p* = 0.023; *r* = 0.449, *p* = 0.006, respectively) in serum cohort-II, while miR-141 was positively correlated with menopausal status (*r* = 0.436, *p* = 0.008) in serum cohort-II. Since the serum cohort-II only consist of early samples, the correlation were nullified during analysis (**Supplementary Table 3**).

### 3.7 Diagnostic Potential of Candidate MiRNAs (From the Tissue Cohort) for Epithelial Ovarian Cancer Prediction

We conducted univariate logistic regression analysis on tissue cohort-I (*n* = 115) and cohort-II (*n* = 71) for each miRNA to retrieve any association of miRNA expression with EOC. The 95% confidence interval was taken as the accuracy of the regression coefficient, and the *p*-value was used to denote statistical significance. The expression level of miR-205, miR-200c, and miR-141 was significantly associated with disease in both tissue cohorts (**Table 2A**).

**Table 2:** Binary logistic regression analysis of tissue {cohort-I [including 44 advance-stage tissue sample (stage III–IV) + 30 normal samples; *n* = 74], cohort-II (including 41 early stage tissue samples + 30 normal samples; *n* = 71)} and serum samples [cohort-I (30 advance-stage III–IV samples + 14 normal samples) (*n* = 44), cohort-II (21 early stage serum samples + 14 normal samples; *n* = 35)].

MiRNA	(A) Tissue cohort (*N* = 115)						(B) Serum cohort (*N* = 65)					
	Cohort-I (stage III–IV) (*n* = 74)			Cohort-II [early stage (stage I + II)] (*n* = 71)			Cohort-I (stage I–IV) (*n* = 44)			Cohort-II [early stage (stage I + II)] (*n* = 35)		
	Regression coefficient (*B*)	95% CI	*p*-value	Regression coefficient (*B*)	95% CI	*p*-value	Regression coefficient (*B*)	95% CI	*p*-value	Regression coefficient (*B*)	95% CI	*p*-value
miR-205	1.144	1.305–7.554	0.011	0.859	1.017–5.477	0.046	1.496	1.116–17.860	0.034	1.534	1.212–17.719	0.025
miR-200c	1.225	1.471–7.873	0.004	0.568	0.388–0.828	0.003	0.618	1.051–3.274	0.033	1.331	0.473–30.28	0.210
miR-141	0.328	1.092–1.763	0.007	3.240	2.890–225.83	0.004	1.134	1.160–8.326	0.024	1.138	0.819–11.906	0.046

Association of outcomes (disease vs. normal) with an increase in the expression of miRNA was determined by regression coefficient (B). The 95% CI was taken as measure of precision of the regression coefficient and statistical significance was determined by p-value. **(A)** In cohort-I of tissue samples, miR-205 (p = 0.011, 95% CI = 1.305–7.554, std error = 0.448), miR-200c (p = 0.004, 95% CI = 1.471–7.873, std error = 0.428), and miR-141 (p = 0.007, 95% CI = 1.092–1.763, std error = 0.122), while in cohort-II, miR-205 (p = 0.046, 95% CI = 1.017–5.477, std error = 0.429), miR-200c (p = 0.003, 95% CI = 0.388–0.828, std error = 0.193), and miR-141 (p = 0.004, 95% CI = 2.890–225.83, std error = 1.111) were significantly associated with disease. **(B)** Similarly, in the serum cohort, miR-205 (p = 0.034, 95% CI = 1.116–17.860, std error = 0.707), miR-200c (p = 0.033, 95% CI = 1.051–3.274, std error = 0.290), and miR-141 (p = 0.024, 95% CI = 1.160–8.326, std error = 0.503) were significantly associated with disease occurrence. In cohort-II of serum, miR-205 (p = 0.025, 95% CI = 1.212–17.719, std error = 0.684) and miR-141 (p = 0.046, 95% CI = 0.819–11.906, std error = 0.683) were showing significant association with disease, while miR-200c did not appear to be statistically significant. All binary logistic regression models were significant with p-value of 0.0001.

B, regression coefficient; CI, confidence interval; p-value, probability value.

**Figure 5:**
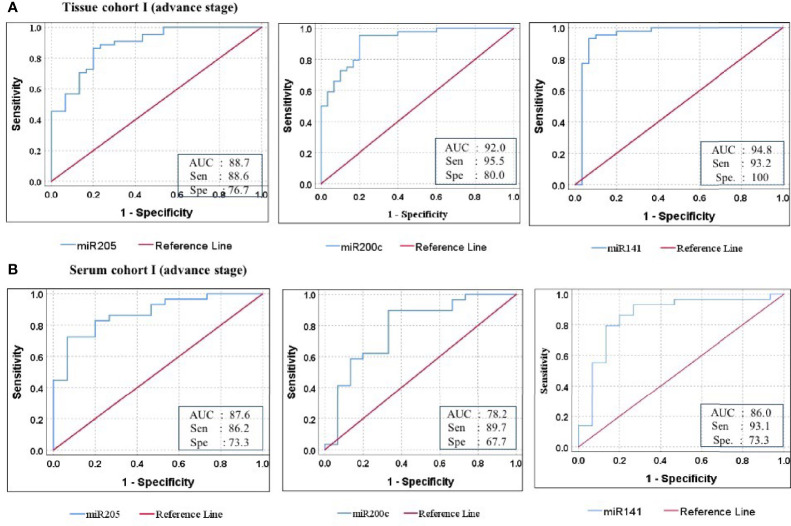
ROC curve analysis in cohort-I of tissue and serum to access the ability of each miRNA signature to diagnose ovarian cancer. **(A)** In tissue cohort-I (*n* = 74), the combined measure of sensitivity and specificity of miR-205, miR-200c, and miR-141 was represented by AUC at 95% CI, *p*-value = 88.7 (0.813–0.961, *p* < 0.001), 92.0 (0.860–0.981, *p* < 0.0001), and 94.8 (0.881–1.016, *p* < 0.0001), respectively. **(B)** In serum cohort-I (*n* = 45), the AUC of miR-205, miR-200c, and miR-141 at 95% CI, *p*-value = 87.6 (0.773–0.979, *p* < 0.001), 78.2 (0.627–0.936, *p* < 0.0001), and 86.0 (0.729–0.990, *p* < 0.0001) respectively. Diagonal reference line acts as a performance measure of the diagnostic test, i.e., whether test yields the negative or positive outcomes by chance or due to relation with the true disease status. AUC, area under the curve; Sen, sensitivity; Sep, specificity.

**Figure 6:**
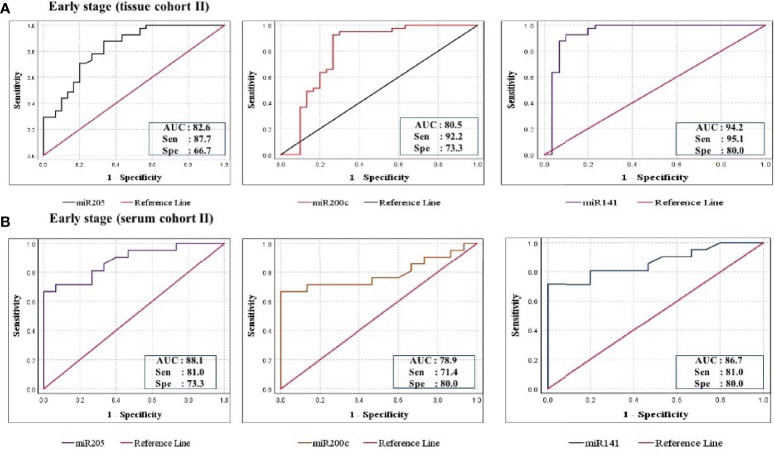
ROC curve analysis in cohort-II of tissue and serum to evaluate the ability of each miRNA signature to diagnose ovarian cancer. **(A)** In tissue cohort-II (*n* = 71), the combined measure of sensitivity and specificity of miR-205, miR-200c, and miR-141 was represented by AUC at 95% CI, *p*-value = 82.6 (0.693–0.928, *p* < 0.0001), 80.5 (0.685–0.925, *p* < 0.0001), and 94.2 (0.873–1.012, *p* < 0.0001), respectively. **(B)** In serum cohort-II (*n* = 35), the AUC of miR-205, miR-200c, and miR-141 at 95% CI, *p*-value = 88.1 (0.773–0.989, *p* < 0.0001), 78.9 (0.636–0.942, *p* < 0.0001), and 86.7 (0.749–0.984, *p* < 0.0001), respectively. AUC, area under the curve; Sen, sensitivity; Sep, specificity.

**Figure 7:**
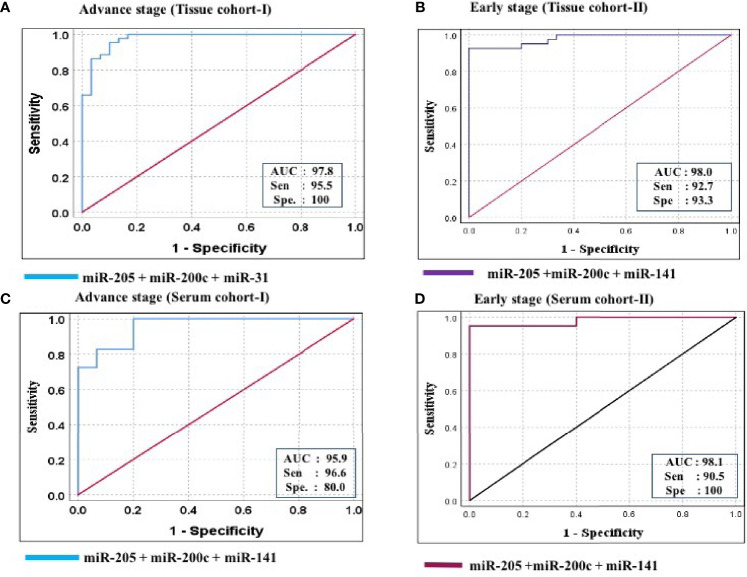
ROC curve analysis in tissue and serum cohort to evaluate the ability of the combined miRNA panel (miR-205 + miR-200c + miR-141) to diagnose ovarian cancer. **(A)** In tissue cohort-I (*n* = 74), the combined measure of sensitivity and specificity of miR-205 + miR-200c + miR-141 was represented by AUC at 95% CI, *p*-value = 97.8 (0.950–1.006, *p* < 0.0001). **(B)** In tissue cohort-II (*n* = 71), the AUC of miR-205 + miR-200c + miR-141 at 95% CI, *p*-value = 98.0 (0.954–1.006, *p* < 0.0001). **(C)** In serum cohort-I (*n* = 45), the combined measure of sensitivity and specificity of miR-205 + miR-200c + miR-141 was represented by AUC at 95% CI, *p*-value = 95.9 (0.925–1.012, *p* < 0.0001) **(D)** In serum cohort-II (*n* = 35), the AUC of miR-205 + miR-200c + miR-141 at 95% CI, *p*-value = 95.2 (0.941–1.021, *p* < 0.0001). Predicted probability, miR-205 + miR-200c + miR-141; AUC, area under the curve; Sen, sensitivity; Sep, specificity.

Furthermore, ROC analysis was performed for individual candidate miRNA in both cohorts of tissue samples. The AUC values of miR-205, miR-200c, and miR-141 in cohort-I were 88.7 (sensitivity = 88.6%, specificity = 76.7%), 92.0 (sensitivity = 95.5%, specificity = 80.0%), and 94.8 (sensitivity = 93.2%, specificity = 100%), respectively (**Figure 5A**). Similarly, in cohort-II, the AUC values of miR-205, miR-200c, and miR-141 were 82.6 (sensitivity = 87.7%, specificity = 66.7%), 80.5 (sensitivity = 92.2%, specificity = 73.3%), and 94.2 (sensitivity = 95.1%, specificity = 80%), respectively (**Figure 6A**). Besides these, a step-wise binary logistic regression analysis was performed to evaluate the combined diagnostic efficiency of the miRNA panel (miR-205, miR-200c, and miR-141) in each tissue cohort to predict the risk of EOC. The combined predicted probability of the three miRNAs from cohort-I and cohort-II was used for ROC analysis. The AUC for the combined miRNA panel in cohort-I and cohort-II was 97.8 (sensitivity = 95.5%, specificity = 100%) and 98.0 (sensitivity = 92.7%, specificity = 93.3%) (**Figures 7A, B**). In both cohorts, individual miRNA and the combined miRNA panel (miR-205 + miR-200c + miR-141) showed better predictive power for epithelial ovarian cancer. The AUC, sensitivity, specificity, and optimum cutoff value of each miRNA are given in **Table 3A**.

**Table 3:** Data of ROC curve analysis for the prediction of epithelial ovarian cancer using the expression level of miRNA in the tissue and serum cohorts, respectively.

MiRNA	(A) Tissue cohort										(B) Serum cohort									
	Cohort-I (stage III–IV)					Cohort-II (stage I–II)					Cohort-I (stage III–IV)					Cohort-II (stage I–II)				
	AUC (%)	SEN (%)	SPE (%)	95% CI	CV	AUC (%)	SEN (%)	SPE (%)	95% CI	CV	AUC (%)	SEN (%)	SPE (%)	95% CI	CV	AUC (%)	SEN (%)	SPE (%)	95% CI	CV
miR-205	88.7	88.6	76.7	0.813–0.961	2.350	82.6	87.7	66.7	0.693–0.928	1.710	87.6	86.2	73.3	0.773–0.979	0.975	88.1	81.0	73.3	0.773–0.989	0.965
miR-200c	92.0	95.5	80.0	0.860–0.981	1.465	80.5	92.2	73.3	0.685–0.925	1.465	78.2	89.7	67.7	0.627–0.936	1.430	78.9	71.4	80.0	0.636–0.942	1.250
miR-141	94.8	93.2	100	0.881–1.016	1.841	94.2	95.1	80.0	0.873–1.012	0.975	86.0	93.1	73.3	0.729–0.990	1.805	86.7	81.0	80.0	0.749–0.984	1.250
Combined	97.8	95.5	100	0.950–1.006	0.515	98.0	92.7	93.3	0.954–1.006	0.490	95.9	96.6	80.0	0.925–1.012	0.401	95.2	90.5	100	0.941–1.021	0.626

**(A)** Evaluation of diagnostic performance of each miRNA in tissue cohort-I and cohort-II. **(B)** Evaluation of diagnostic performance of each miRNA and combined miRNA panel in serum cohort-I and cohort-II.

AUC, area under the curve; SEN, sensitivity; SEP, specificity; CI, confidence interval; CV, optimum cut-off values; Combined, cumulative predicted probability of miR-205+ miR-200c + miR-141.

### 3.8 Diagnostic Potential of Candidate MiRNAs (From the Serum Cohort) for Epithelial Ovarian Cancer Prediction

The consistency of diagnostic performance of individual miRNA and combined miRNA panel was validated in the serum cohort (50 EOC and 15 normal). The binary logistic regression model revealed a significant association of miR-205, miR-200c, and miR-141 with disease occurrence (**Table 2B**).

Further ROC analysis was conducted for individual miRNA from the serum cohort. The AUC values of miR-205, miR-200c, and miR-141 in cohort-I were 87.6 (sensitivity = 86.2%, specificity = 73.3%), 78.2 (sensitivity = 89.7%, specificity = 67.2%), and 86.0 (sensitivity = 93.1%, specificity = 73.3%), respectively (**Figure 5B**). Similarly, in cohort-II, the AUC values of miR-205, miR-200c, and miR-141 were 88.1 (sensitivity = 81%, specificity = 73.3%), 78.9 (sensitivity = 71.4%, specificity = 80%), and 86.7 (sensitivity = 81%, specificity = 80%), respectively (**Figure 6B**). The combined AUC for cohort-I and cohort-II was 95.9 (sensitivity = 96.6%, specificity = 80.0%) and 95.2 (sensitivity = 90.5%, specificity = 100%), respectively (**Figures 7C, D**). In both tissue and serum cohorts, miR-205 and miR-141 turned out to be the best performing expression markers for ovarian cancer prediction at an early stage. The combined miRNA panel from cohort-I and cohort-II has shown much higher AUC, sensitivity, and specificity than the single marker for predicting EOC. In addition, the diagnostic performance of individual miRNA and miRNA panels from serum was consistent with the diagnostic performance of miRNA from tissue cohorts. The AUC, sensitivity, specificity, and optimum cutoff value of each miRNA are given in **Table 3B**.

### 3.9 MiRNA–Target Prediction and MiRNA–Disease Enrichment Analysis

**Figure 8:**
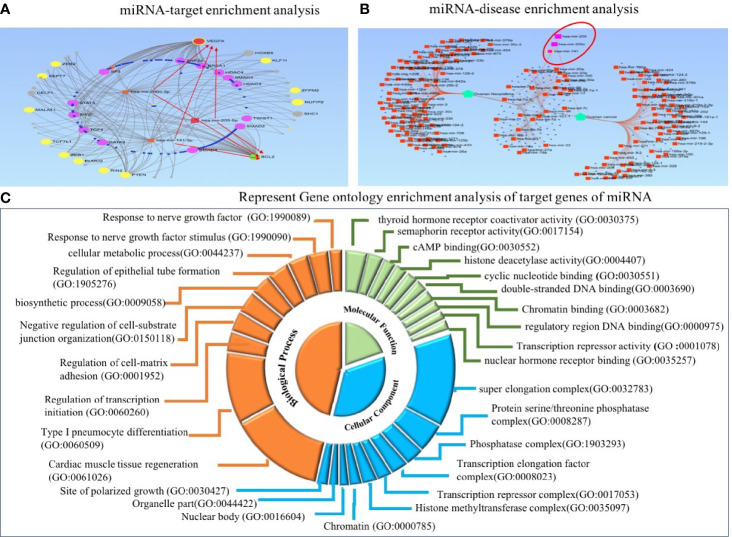
Target enrichment and Gene Ontology enrichment analysis of miRNA–target genes. **(A)** In regulatory network analysis, VEGFA and BCL2 act as central target molecules of three candidate miRNAs. **(B)** MiRNA–disease enrichment analysis revealed candidate miRNAs were associated with ovarian cancer disease. **(C)** In Gene Ontology analysis, the involvement of three miRNAs in several important biological, molecular, and cellular processes was revealed.

To explore the functional role of dysregulated candidate miRNAs in ovarian cancer, we performed screening of target genes of candidate miRNA using six online tools (miRDB, Tools4miRS, TargetScanHuman7.2, miRWalk2.0, miRanda, RNAhybrid). More than 1,000 genes were targeted by individual miRNA from each database; however, after manual sorting of genes from six online databases, we found 396 target genes overlapping in all databases (**Supplementary Table 4**). Furthermore, these candidate miRNA–target interactions were used to build a miRNA–target regulatory network using miRNet 2.0 with default parameters. The filtering used in miRNet 2.0 allowed us to only connect three miRNA with target genes. In the regulatory network, *VEGFA* and *BCL2* were highlighted to be the central target genes of miR-205, miR-200c, and miR-141 and shown in the red arrow. In addition, *ZEB1*, *ZEB2*, *PTEN*, and *SEPT7* were regulated by the three candidate miRNAs, and many studies confirm the prominent role of these genes in several biological processes and cancer progression (**Figure 8A**). Furthermore, we performed miRNA–disease enrichment analysis using miRNet 2.0 tools with default parameters (ovarian cancer/neoplasm). A total of 349 miRNAs were associated with ovarian cancer with 487 edges, including our selected candidate miRNA (**Figure 8B**). This analysis revealed the importance of candidate miRNA in regulating different tumor-suppressor genes in the biological system.

#### 3.9.1 Functional Enrichment Analysis of MiRNA–Target Genes

Predicted target genes were further used to perform GO enrichment analysis and KEGG pathway enrichment analysis. Gene Ontology analysis was used to acknowledge the systemized hallmark and biological meaning of target genes using the GO stat package. The top 10 Gene Ontology terms for each category, including biological process (BP), molecular function (MF), and cellular component (CC), were enriched for candidate miRNA–target genes and presented as a doughnut pie chart in **Figure 8C**, which include histone methyltransferase complex, chromatin, nuclear body, and organelle part in CC categories; cyclic nucleotide binding and histone deacetylase activity in MF categories; and regulation of transcription initiation, biosynthetic process, and cellular metabolic process in BP categories (**Figure 8C**).

In addition, we performed KEGG pathway enrichment analysis of target genes to investigate miRNA-regulated pathways that could reveal the underlying process of ovarian cancer by using the DAVID database. The pathways enriched with candidate miRNA–targets were reportedly involved in metastasis, invasion, and cancer progression; these are transcriptional misregulation in cancer, Wnt signaling pathway, mTOR signaling pathway, MAPK signaling pathway, and miRNA in cancer. All pathways were significantly enriched by miRNA–target genes with a *p*-value <0.05 (**Table 4**). Furthermore, to explore the importance of these enriched pathways in cancer, we performed PubMed search for published articles relating the roles of the top 7 pathways in cancer (**Supplementary Table 5**).

**Table 4:** Top 16 KEGG pathway enrichment for targets of candidate miRNA.

Term	Count	%	*p*-value	Pop hits	Pop total	Fold enrichment	Bonferroni	Benjamini	FDR
hsa05202: transcriptional misregulation in cancer	10	2.76243094	0.00307618	167	6,879	3.295329341	0.420345275	0.15947673	0.15677374
hsa05205: proteoglycans in cancer	11	3.03867403	0.00315527	200	6,879	3.02676	0.428429023	0.15947673	0.15677374
hsa04310: Wnt signaling pathway	9	2.48618785	0.00333975	138	6,879	3.589043478	0.446849298	0.15947673	0.15677374
hsa04114: oocyte meiosis	8	2.20994475	0.00376449	111	6,879	3.96627027	0.487047825	0.15947673	0.15677374
hsa05200: pathways in cancer	16	4.4198895	0.00450499	393	6,879	2.24048855	0.550304767	0.15947673	0.15677374
hsa04071: sphingolipid signaling pathway	8	2.20994475	0.00577366	120	6,879	3.6688	0.641167652	0.17032307	0.16743624
hsa04340: Hedgehog signaling pathway	4	1.10497238	0.01219625	27	6,879	8.152888889	0.886051388	0.30839078	0.30316382
hsa04010: MAPK signaling pathway	11	3.03867403	0.0156372	253	6,879	2.392695652	0.938557416	0.34101917	0.33523918
hsa04919: thyroid hormone signaling pathway	7	1.93370166	0.01733996	115	6,879	3.349773913	0.954774484	0.34101917	0.33523918
hsa04150: mTOR signaling pathway	5	1.38121547	0.02018706	58	6,879	4.744137931	0.972939181	0.35731102	0.3512549
hsa04015: Rap1 signaling pathway	9	2.48618785	0.03548154	210	6,879	2.358514286	0.998329038	0.57093016	0.56125338
hsa04390: Hippo signaling pathway	7	1.93370166	0.0447634	151	6,879	2.551152318	0.999953154	0.80776021	0.79406936
hsa04727: GABAergic synapse	5	1.38121547	0.06682952	85	6,879	3.237176471	0.999995179	0.90990967	0.89448747
hsa05206: microRNAs in cancer	10	2.76243094	0.04257739	286	6,879	1.924195804	0.999998385	0.91758551	0.90203322
hsa04728: dopaminergic synapse	6	1.65745856	0.08091666	128	6,879	2.579625	0.999999674	0.95481662	0.93863328

## 4 Discussion

The advancement of OC is a consequence of the multistep dysregulated function of oncogenes and tumor-suppressor genes (11). The higher morbidity rate of ovarian cancer is the combined consequence of failure in early detection and therapeutic interventions (26). CA125 is the most commonly used serum biomarker for the detection of OC; however, its effectiveness in diagnosing early-stage ovarian cancer is still debatable (27–30). Therefore, these limitations of the current serum-based diagnostic biomarker urge us to identify a new promising biomarker for early detection of EOC for better management and prognosis of the disease.

A large group of studies showed the aberrant expression of miRNA in different types of cancer, such as prostate, breast, and ovarian cancer (31–34). Besides this, several studies have explored the significance of circulatory miRNAs as a diagnostic biomarker for cancer (35). Regardless of the ribonucleases in the blood, circulatory miRNAs are highly stable because they are packed in exosome or apoptotic bodies, making them resistant to degradation (36, 37). Therefore, miRNA expression studies conducted on liquid biopsies from cancer patients may lead to establishing circulating miRNA as potential signatures for ovarian cancer detection.

As the first step toward identifying robust candidate miRNAs for early diagnosis of ovarian cancer, we selected three hypomethylated miRNAs (miR-205, miR-200c, and miR-141) from MeDIP-NGS sequencing data analysis of EOC samples (**Supplementary Table 2**). We then explored their relative expression in tissue and serum cohorts of EOC patients and compared it with normal control. In our study, the mean expression levels of all individual miRNAs were significantly elevated in both tissue and serum cohorts of EOC samples compared with those of normal control samples. In addition to this, we also explored the miRNA expression patterns in early-stage EOC samples (stage I–II); we found significant elevation of miRNAs in both the tissue and serum cohort as compared with that in normal control. Our finding was consistent with the previous study by Loginov et al. showing hypomethylation of miRNA promoter, which could be the possible mechanism responsible for elevated expression of miRNA in OC (15). Similarly, Iorio et al. established that elevation of miR-205 in EOC was coupled with hypomethylation of miRNA promoter (38). Davalos et al. demonstrated the hypomethylation of CpG island of miR-141/200c in cancer cell with epithelial features (39, 40).

Moreover, aberrant expressions of the selected candidate miRNAs and their role in cancer progression were evident in several recent studies. Wei et al. reported that miR-205, a key regulator of TCF21, was frequently elevated; inhibition of TCF21 by miR-205 leads to overexpression of MMP10 (key player of metastasis and cell invasion), which further promotes ovarian cancer progression, metastasis, and invasion (41). In another study, Li et al. revealed that enhanced levels of miR-205 deregulate SMAD4 and PTEN (a central molecule of the TGF-B signaling pathway), leading to enhanced cell proliferation in OC (42). Similarly, increased expression of the miR-200 family was found to be involved in ovarian cancer induction and metastasis (43–45). In addition, an *in-vitro* study reported miR-141 elevation in SOC tissue and cell lines compared with that in normal, and inhibition of miR-141 enhances the expression of DLC-1 and ZEB2 leading to migration and metastases (46). Ibrahim et al. found significant elevation of miR-200c in ovarian cancer; *in-vitro* transfection analysis revealed that inhibition of miR-200c suppresses DLC-1 level and enhances cell proliferation (47). Conferring these studies, our finding suggests that the elevated expression level of miR-205, miR-200c, and miR-141 might be involved in cancer progression by targeting cancer-associated tumor-suppressor genes and might help in discriminating EOC patients from normal controls (45, 48–50).

The expression-specific candidate miRNA in ovarian cancer undertaken in this study has been previously documented. In contrast, reduced expression of these miRNAs has been documented in other cancer types. Downregulated expression of miR-205 has been reported in breast cancer, prostate cancer, pancreatic cancer, renal cancer, and thyroid cancer (51–56). Similarly, there was downregulated expression of miR-141 in breast cancer, bone metastases, prostate cancer, and renal cancer tissue and cell lines, and pancreatic cancer were reported (57–61). A recent study by Rahimi et al. reported downregulated expression of miR-200c in breast cancer cell line and tumor tissue compared with that in control (62). Moreover, miR-200c downregulation has been seen in prostate cancer, pancreatic cancer, renal cancer, and thyroid carcinoma (63–67). We further examined the diagnostic performance of individual and combined miRNA panels in tissue and serum cohorts. Our finding on tumor tissue suggests that miR-205, miR-200c, and miR-141 could diagnose EOC with higher AUC, sensitivity, and specificity. When a similar analysis was performed in serum cohorts, miR-200c exhibited slightly lower diagnostic ability in terms of AUC and sensitivity compared with miR-205 and miR-141. The combined diagnostic performance of all three miRNAs from the tissue and serum cohorts has shown higher AUC, sensitivity, and specificity values for early EOC prediction as well. These finding suggests that the combined serum miRNA panel could be used as a minimally invasive early diagnostic biomarker for epithelial ovarian cancer. However, the individual diagnostic performance of miR-205 and miR-141 is comparable with the combined marker panel. Similarly, a recent study by Wang et al. identified a miRNA panel that was significantly elevated in OC, and miR-205 could predict ovarian cancer with an AUC value of 0.681 (68). Gao et al. identified two miRNAs (miR-200c and miR-141) that were significantly overexpressed in OC and could diagnose ovarian cancer patients with AUC of 0.79 and 0.75, respectively (49). The combined miRNA marker panel in the present study showed better diagnostic value for advance grade and early epithelial ovarian cancer prediction.

Furthermore, we tried to link miRNA expression profile with clinical characteristics of EOC. We attempted to classify the histopathological subtype of EOC based on miRNA expression in tissue samples. Elevated miR-205 was positively associated with mucinous histotype, while miR-200c and miR-141 were significantly associated with endometrioid and serous histotypes. However, expression profiling in serum did not show any significant association with histological subtype. Moreover, the expression of the three miRNAs could be further helpful in the cancer staging system and metastasis status. Advance stages (stage III–IV) and the metastatic nature of epithelial ovarian cancer were associated with the higher expression of these candidate miRNAs. In addition, the expression level of candidate miRNAs showed a positive correlation with serum CA125, menopausal status, and distant metastasis. However, an association of miRNA expression with these clinical features should be evaluated at the molecular level in the future for a better understanding of disease progression.

In addition, we performed miRNA–disease enrichment, target enrichment network analysis, and function enrichment analysis (GO and KEGG) of predicted target genes of the candidate miRNA. MiRNA–disease enrichment analysis revealed the association of candidate miRNA with ovarian cancer (69, 70). Similarly, miRNA–target gene regulatory network analysis revealed *VEGFA* and *BCL2* as central target molecules, and genes like *ZEB1*, *ZEB2*, *PTEN*, and *SEPT7* were found to have importance in several biological pathways. Moreover, the top 10 enriched Gene Ontology terms are important at the biological, molecular, and cellular levels. Moreover, several important pathways such as Wnt, mTOR, *MAPK* signaling, and miRNA in cancer were significantly enriched in KEGG pathway enrichment analysis (71–75). Recent reports suggest that Wnt signaling pathway regulates several crucial events, including EMT, cell migration, cell proliferation, and polarity of cells (76–81). The association of Wnt signaling pathway and dysregulation of miRNA has been linked together in various cancer types. Similarly, the mTOR signaling pathway exerts regulation of cell proliferation, angiogenesis, metabolism, and apoptosis. mTOR upstream or downstream cascade molecules were often targeted by dysregulated miRNA and promoted cancerous phenotypes. PTEN, a key molecule of P13K/Akt/mTOR signaling pathway, has been shown to be inhibited by miR-205, miR-200c, and miR-141 in several other cancers (10, 82–86). In addition, MAPK signaling-based regulation of cellular proliferation, cellular differentiation, and death was also found to be regulated by candidate miRNAs in several cancers (87–89).

Overall, our study establishes a proof of concept that miRNA gene hypomethylation and their downstream expression are correlated, and the aberrant expression of these miRNA has the potential to diagnose ovarian cancer at an early stage. In addition, to the best of our knowledge, this is the first report implicating the combined predictive power of miR-205, miR-200c, and miR-141 in early diagnosis of epithelial ovarian cancer with higher sensitivity and specificity. Since our samples are restricted only to the north Indian population, samples from other demographic areas need to be included to further validate these findings in a larger cohort of samples. Nevertheless, a diagnostic test based on circulatory miRNA expression has to overcome several hurdles before its clinical implementation as a biomarker. Success is entirely dependent upon the universal protocol for miRNA isolation, normalization, and effective quantitative expression analysis technique at a low cost.

## Data Availability Statement

The original contributions presented in the study are publicly available. These data can be found here: National Center for Biotechnology Information (NCBI) BioProject database under accession number GSE180292.

## Ethics Statement

The studies involving human participants were reviewed and approved by the Institute Ethical Committee, Motilal Nehru National Institute of Technology. The patients/participants provided their written informed consent to participate in this study.

## Author Contributions

VK executed the experiments, validated the results, and wrote the manuscript. SG provided the samples and executed the statistical analysis. AC provided the samples and was responsible for manuscript preparation. MS designed the study and was responsible for proofreading, validation of the results, and publishing the article. All authors contributed to the article and approved the submitted version.

## Funding

This research received a specific grant from the Indian Council of Medical Research, Government of India, Grant number 5/13/58/2015/NCD-III.

## Conflict of Interest

The authors declare that the research was conducted in the absence of any commercial or financial relationships that could be construed as a potential conflict of interest.

## Publisher’s Note

All claims expressed in this article are solely those of the authors and do not necessarily represent those of their affiliated organizations, or those of the publisher, the editors and the reviewers. Any product that may be evaluated in this article, or claim that may be made by its manufacturer, is not guaranteed or endorsed by the publisher.

## Supplementary Material

Supplementary Figure 1 Expression analysis of hypomethylated miRNA in samples used for MeDIP-NGS analysis (six EOC and two normal samples). miR-205, miR-200c and miR-141 was significantly elevated in cancer compared to control with fold change of 3.85 (p < 0.05), 4.95 (p < 0.05), and 4.69 (p < 0.05), respectively.

Supplementary Figure 2 Represents expression of miRNA in different FIGO stages of epithelial ovarian cancer. **(A)** In tissue cohort {n=85 (41 stage-I+II and 44 stage-III+IV)}, expression levels of miR-205, miR-200c and 141 were significantly elevated in combined stage III-IV (P = 0.05; P < 0.005; P < 0.005 respectively), **(B)** In serum cohort {n=51 (21 stage-I+II and 30 stage-III+IV)}, relative expression of miR-205, miR-200c and miR-141 was significantly elevated in stage III-IV (P < 0.05, P < 0.005, P < 0.005 respectively). Statistically significant differences were determined by the One-way ANOVA test and Mann Whitney U-tests. Data represent mean ± standard error on the mean (SEM). *P < 0.05; **P < 0.01; ***P < 0.0001; ns, not significant.

Supplementary Figure 3 Represents expression of miRNAs in different histotypes of epithelial ovarian cancer. **(A)** In tissue cohort (n=85 cancerous sample), expression levels of miR-205 was able to significantly discriminate mucinous from other subtypes of EOC (P <0.005). Similarly, miR-200c was significantly elevated in endometrioid subtype (p < 0.005) while, miR-141 significantly elevated in serous subtypes (p < 0.05) to compared histotypes **(B)** In serum cohort (n=45 cancerous sample), relative expression of miR-205, miR-200c and miR-141 were not changed in subtype EOC. Statistically significant differences were determined by the One-way ANOVA test. Data represent mean ± standard error on the mean (SEM). *P < 0.05; **P < 0.01; ***P < 0.0001; ns, not significant. Ser, serous; Muc, Mucinous; CC, Clear cell; Endo, Endometrioid subtype.

Supplementary Figure 4 Represents expression of miRNA in metastatic and non-metastatic samples of epithelial ovarian cancer. **(A)** In tissue cohort {n=85 (44 metastatic and 41 non-metastatic)}, expression levels of miR-205, miR-200c and 141 were significantly discriminating non-metastatic and metastatic cases (P <0.0001; P <0.0001; P <0.0001 respectively). **(B)** Similarly, in serum cohort {n=55 (30 metastatic and 21 non-metastatic)}, relative expression of miR-205, miR-200c and miR-141 was significantly elevated in metastatic cases as compare to non-metastatic cases (P <0.001; P <0.001, P <0.001 respectively). Statistically significant differences were determined by the Mann Whitney U-tests. Data represent mean ± standard error on the mean (SEM). *P < 0.05; **P < 0.01; ***P < 0.0001; ns, not significant; NM, Non-metastatic; M, metastatic.

## Abbreviations

OC, ovarian cancer; EOC, epithelial ovarian cancer; MeDIP, methylated DNA immunoprecipitation; miR or miRNA, microRNA; ROC, receiver operating characteristics; AUC, area under the curve; CA125, cancer antigen 125; FIGO, International Federation of Gynaecology and Obstetrics; qRT-PCR, real-time quantitative reverse transcription PCR; EMT, epithelial–mesenchymal transition; GO, Gene Ontology; KEGG, Kyoto Encyclopedia of Genes and Genomes.
