# Supplementary figures and images for: Evaluation of Diagnostic Potential of Epigenetically Deregulated MiRNAs in Epithelial Ovarian Cancer
Source: Front Oncol. 2021 Oct 7;11:681872. doi: 10.3389/fonc.2021.681872 (PMC8529058; doi:10.3389/fonc.2021.681872)

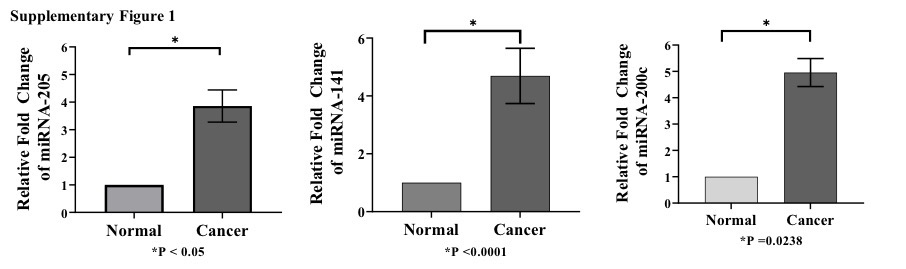

Supplement: Supplementary Figure 1 — Expression analysis of hypomethylated miRNA in samples used for MeDIP-NGS analysis (six EOC and two normal samples). miR-205, miR-200c and miR-141 was significantly elevated in cancer compared to control with fold change of 3.85 (p < 0.05), 4.95 (p < 0.05), and 4.69 (p < 0.05), respectively. [file Image_1.jpeg]

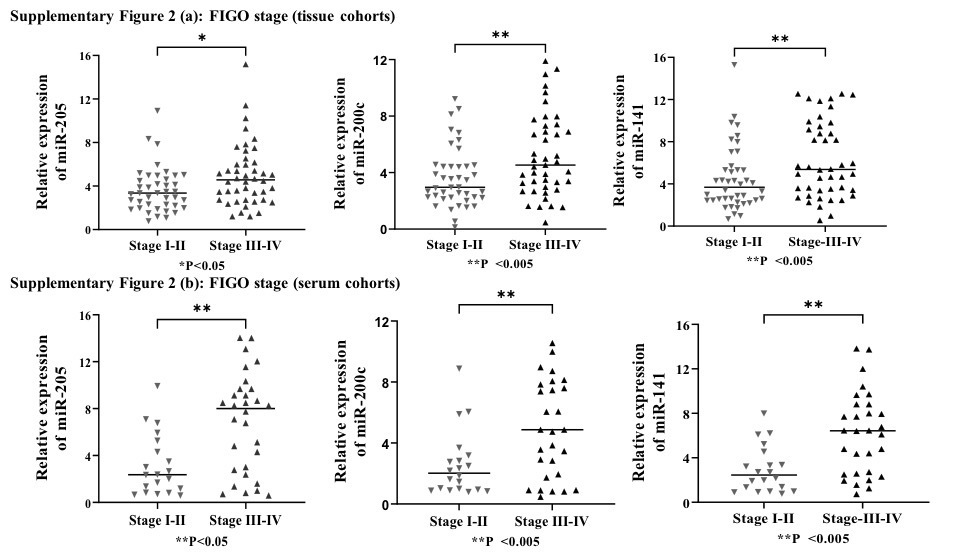

Supplement: Supplementary Figure 2 — Represents expression of miRNA in different FIGO stages of epithelial ovarian cancer. (A) In tissue cohort {n=85 (41 stage-I+II and 44 stage-III+IV)}, expression levels of miR-205, miR-200c and 141 were significantly elevated in combined stage III-IV (P = 0.05; P < 0.005; P < 0.005 respectively), (B) In serum cohort {n=51 (21 stage-I+II and 30 stage-III+IV)}, relative expression of miR-205, miR-200c and miR-141 was significantly elevated in stage III-IV (P < 0.05, P < 0.005, P < 0.005 respectively). Statistically significant differences were determined by the One-way ANOVA test and Mann Whitney U-tests. Data represent mean ± standard error on the mean (SEM). *P < 0.05; **P < 0.01; ***P < 0.0001; ns, not significant. [file Image_2.jpeg]

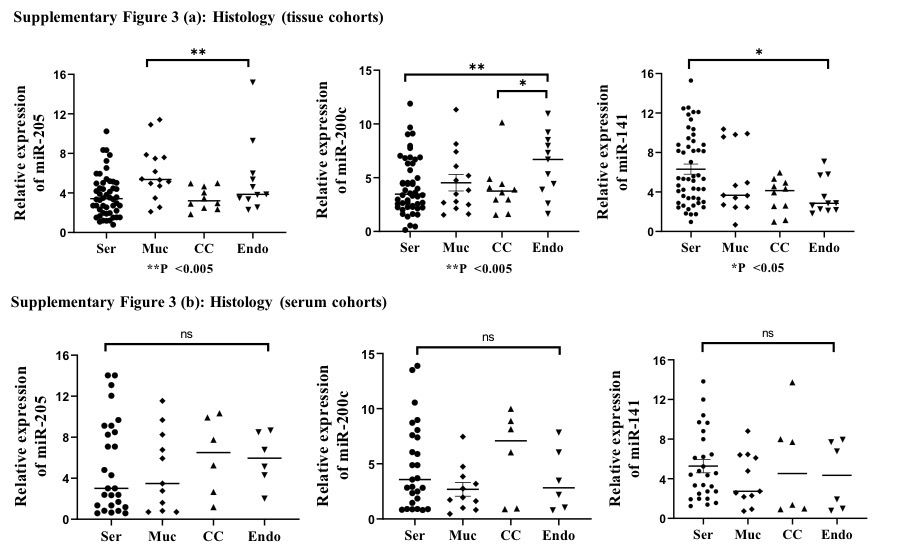

Supplement: Supplementary Figure 3 — Represents expression of miRNAs in different histotypes of epithelial ovarian cancer. (A) In tissue cohort (n=85 cancerous sample), expression levels of miR-205 was able to significantly discriminate mucinous from other subtypes of EOC (P <0.005). Similarly, miR-200c was significantly elevated in endometrioid subtype (p < 0.005) while, miR-141 significantly elevated in serous subtypes (p < 0.05) to compared histotypes (B) In serum cohort (n=45 cancerous sample), relative expression of miR-205, miR-200c and miR-141 were not changed in subtype EOC. Statistically significant differences were determined by the One-way ANOVA test. Data represent mean ± standard error on the mean (SEM). *P < 0.05; **P < 0.01; ***P < 0.0001; ns, not significant. Ser, serous; Muc, Mucinous; CC, Clear cell; Endo, Endometrioid subtype. [file Image_3.jpeg]

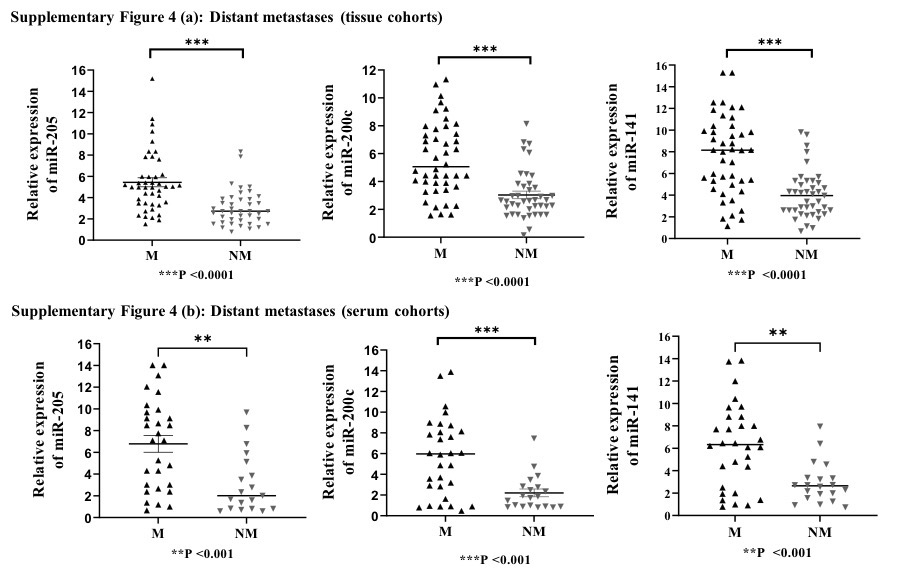

Supplement: Supplementary Figure 4 — Represents expression of miRNA in metastatic and non-metastatic samples of epithelial ovarian cancer. (A) In tissue cohort {n=85 (44 metastatic and 41 non-metastatic)}, expression levels of miR-205, miR-200c and 141 were significantly discriminating non-metastatic and metastatic cases (P <0.0001; P <0.0001; P <0.0001 respectively). (B) Similarly, in serum cohort {n=55 (30 metastatic and 21 non-metastatic)}, relative expression of miR-205, miR-200c and miR-141 was significantly elevated in metastatic cases as compare to non-metastatic cases (P <0.001; P <0.001, P <0.001 respectively). Statistically significant differences were determined by the Mann Whitney U-tests. Data represent mean ± standard error on the mean (SEM). *P < 0.05; **P < 0.01; ***P < 0.0001; ns, not significant; NM, Non-metastatic; M, metastatic. [file Image_4.jpeg]
