# Supplementary material for: Evaluation of Diagnostic Potential of Epigenetically Deregulated MiRNAs in Epithelial Ovarian Cancer
Source: Front Oncol. 2021 Oct 7;11:681872. doi: 10.3389/fonc.2021.681872 (PMC8529058; doi:10.3389/fonc.2021.681872)
Supplement: Supplementary file 5 [file Table_1.docx]

| Sample Number | Total number of bases | Total number of cleaned reads | Total number of mapped reads | Mapping percentage |
| --- | --- | --- | --- | --- |
| N65 | 10.68 Gb | 144,616,084 | 133,298,461 | 92.17% |
| N66 | 12.02 Gb | 160,104,868 | 148,376,154 | 92.67% |
| T55 | 09.38 Gb | 124,953,164 | 116,217,593 | 93.01% |
| T56 | 10.99 GB | 146,356,270 | 136,078453 | 92.98% |
| B10 | 08.69 GB | 62,202,086 | 49,957,304 | 80.30% |
| T65 | 08.97 GB | 63,761,008 | 53,917,982 | 84.56% |
| T21 | 08.35 GB | 66,604,730 | 56,519,191 | 84.85% |
| T28 | 09.08 GB | 61,520,612 | 53,916,362 | 87.63% |

**Supplementary Table I:** Represent MeDIP NGS mapping rate of six EOC and two normal samples.
