# Supplementary material for: Evaluation of Diagnostic Potential of Epigenetically Deregulated MiRNAs in Epithelial Ovarian Cancer
Source: Front Oncol. 2021 Oct 7;11:681872. doi: 10.3389/fonc.2021.681872 (PMC8529058; doi:10.3389/fonc.2021.681872)
Supplement: Supplementary file 6 [file Table_2.docx]

| **miRNA** | **Chr** | **Meth. Start** | **Meth. End** | **miRNA Prom Start** | **miRNA Prom End** | **#Bases over** | **OC-Avg.** | **C-Avg.** | **Event** | **log2FC** | **pVal** | **Gene Symbol** | **Transcript Name** | **TSS** | **TES** | **Feature** |
| --- | --- | --- | --- | --- | --- | --- | --- | --- | --- | --- | --- | --- | --- | --- | --- | --- |
| hsa-mir-429 | chr1 | 1156701 | 1158400 | 1157942 | 1162941 | 458 | 253.28 | 295.02 | Down | -0.22 | 0.012 | SDF4 | NM_016176 | 1152287 | 1167447 | GB |
| hsa-mir-200b | chr1 | 1156701 | 1158400 | 1157942 | 1162941 | 458 | 253.28 | 295.02 | Down | -0.22 | 0.012 | SDF4 | NM_016176 | 1152287 | 1167447 | GB |
| hsa-mir-9-1 | chr1 | 156432001 | 156433100 | 156429015 | 156434014 | 1099 | 84.33 | 103.56 | Down | -0.3 | 0.047 | MEF2D | NM_005920 | 156433518 | 1.56E+08 | GB |
| **hsa-mir-205** | **Chr1** | **209427140** | **209435965** | **209433601** | **209432200** | **999** | **139.52** | **186.21** | **Down** | **-0.21** | **0.002** | **NA** | **NA** | **NA** | **NA** | **OI** |
| **hsa-mir-200c** | **chr12** | **6926101** | **6927600** | **6922814** | **6927813** | **1499** | **132.59** | **156.31** | **Down** | **-0.24** | **0.048** | **CD4** | **NM_001195014** | **6898637** | **6929976** | **GB** |
| **hsa-mir-141** | **chr12** | **6926101** | **6927600** | **6922814** | **6927813** | **1499** | **132.59** | **156.31** | **Down** | **-0.24** | **0.048** | **CD4** | **NM_001195014** | **6898637** | **6929976** | **GB** |
| hsa-mir-134 | chr14 | 101018901 | 101020400 | 101020103 | 101025102 | 297 | 129.63 | 148.24 | Down | -0.19 | 0.114 | BEGAIN | NM_001159531 | 101003483 | 1.01E+08 | GB |
| hsa-mir-338 | chr17 | 81131101 | 81132100 | 81128880 | 81133879 | 999 | 76.1 | 97.49 | Down | -0.36 | 0.021 | NA | NA | NA | NA | OI |
| hsa-mir-99b | chr19 | 51684201 | 51686000 | 51685021 | 51690020 | 979 | 225.8 | 262.24 | Down | -0.22 | 0.020 | NA | NA | NA | NA | OI |
| hsa-mir-125a | chr19 | 51684201 | 51686000 | 51685066 | 51690065 | 934 | 225.8 | 262.24 | Down | -0.22 | 0.020 | NA | NA | NA | NA | OI |
| hsa-mir-149 | chr2 | 240449401 | 240450400 | 240447692 | 240452691 | 999 | 67.54 | 85.11 | Down | -0.33 | 0.044 | NA | NA | NA | NA | OI |
| hsa-let-7g | chr3 | 52278801 | 52280900 | 52277041 | 52288361 | 2099 | 184.43 | 235.04 | Down | -0.35 | 0.000 | PPM1M | NM_144641 | 52279808 | 52284615 | PI |
| hsa-mir-340 | chr5 | 180040901 | 180042000 | 180040299 | 180045298 | 1099 | 100.71 | 123.27 | Down | -0.29 | 0.033 | FLT4 | NM_002020 | 180034752 | 1.8E+08 | GB |
| hsa-mir-491 | chr9 | 20679701 | 20681300 | 20679263 | 20684262 | 1599 | 81.44 | 99.89 | Down | -0.29 | 0.052 | KIAA1797 | NM_017794 | 20658308 | 20995954 | GB |
| hsa-mir-126 | chr9 | 136664601 | 136666400 | 136664262 | 136669261 | 1799 | 130.31 | 169.33 | Down | -0.38 | 0.001 | VAV2 | NM_003371 | 136627015 | 1.37E+08 | GB |

Supplementary Table 2. Top 15 miRNA showing hypomethylation in six EOC cases (average of six OC sample) as compare to normal (average of two normal sample) as revealed by MeDIP NGS sequencing. There miRs were sorted on the basis of log2FC and P-Value. GB: Gene body; OI: Other Intergenic region, PP: Proximal promoter, OC Avg: Ovarian cancer average, C-Avg: Control Average, Meth : Methylation, TSS: Transcription Start Site, TES: Transcription End Site , Chr: Chromosome; Highlighted rows represents selected miRNA for study.
