# Supplementary material for: Evaluation of Diagnostic Potential of Epigenetically Deregulated MiRNAs in Epithelial Ovarian Cancer
Source: Front Oncol. 2021 Oct 7;11:681872. doi: 10.3389/fonc.2021.681872 (PMC8529058; doi:10.3389/fonc.2021.681872)
Supplement: Supplementary file 7 [file Table_3.docx]

| 1. Tissue Cohort | | | | | | | 1. Serum Cohort | | | | | | |  |
| --- | --- | --- | --- | --- | --- | --- | --- | --- | --- | --- | --- | --- | --- | --- |
| Cohort-I (stage III-IV) | | | | Cohort-II (stage I-II) | | | Cohort-I (stage III-IV) | | | Cohort-II (stage I-II) | | | |  |
| Parameters |  | miR-205 | miR-200c | miR-141 | miR-205 | miR-200c | miR-141 | miR-205 | miR-200c | miR-141 | miR-205 | miR-200c | miR-141 |  |
| Age | | r  P | 0.074  0.532 | 0.079  0.504 | 0.007 0.951 | 0.046 0.706 | 0.206 0.085 | 0.134 0.264 | **0.477 0.001** | 0.083 0.595 | **0.330 0.031** | 0.104 0.552 | -0.160 0.359 | 0.030 0.864 |
| CA125 | | r  P | **0.380**  **0.001** | **0.458 0.0001** | **0.428**  **0.0001** | 0.200 0.095 | 0.164 0.171 | **0.349 0.003** | **0.455 0.002** | **0.570 0.0001** | **0.471 0.001** | **0.483 0.003** | **0.378 0.023** | **0.449 0.006** |
| Menopausal  Status | | r  P | 0.026  0.826 | 0.119 0.311 | 0.116 0.327 | 0.092 0.446 | **0.242 0.042** | 0.183 0.127 | **0.617 0.0001** | **0.463 0.002** | **0.591 0.0001** | 0.162 0.346 | 0.318 0.059 | **0.436 0.008** |
| Distant metastases | | r  P | **0.353 0.002** | **0.417 0.0001** | **0.333 0.004** | 0.045 0.407 | 0.200 0.995 | 0.182 0.129 | **0.462 0.002** | **0.357 0.017** | **0.484**  **0.001** | -0.082 0.633 | -0.058 0.737 | -0.044 0.801 |

**Supplementary Table 3**. Represents correlation of miRNAs with clinical characteristics in tissue and serum cohort of EOC. Cohort-I consist of advance stage EOC samples (stage III - stage IV) and cohort-II consist of stage I-II EOC samples. A) miR-205, miR-200c and miR-141 were positively correlated with distant metastases and CA125 in tissue cohort-I with r=0.353; p<0.002, r=0.417; p <0.0001, r=0.333; p <0.004 and r=0.380; p=0.001, r=0.458; p =0.0001, r=0.428; p <0.0001 respectively. In tissue cohort-II, miR-141 was positively correlated with CA125 with r=0.349; p<0.003 and miR-200c was significantly correlated with menopausal status in cohorts-II. While distance metastases were not correlated with any tested miRNA (B) Represents correlation of serum miRNA with clinical characteristics. miR-205, miR-200c and miR-141 were positively correlated with CA125, menopausal status and distance metastases in serum cohort-I. while, miRNA-205 and miRNA-141 was positively correlated with age in serum cohort-I except miRNA-200c. In serum cohort-II, miRNA-205, -200c and miRNA-141 was positively correlated with CA125 level (r=0.483, p=0.003; r=0.378, p=0.023; r=0.449, p=0.006, respectively). In addition, miRNA-141 have strong positive correlation with menopausal status. Statistical significance was determined by using Sperarman rank correlation coefficient analysis. r= correlation coefficient, bold characters: statistically significant values, P: P-value.
