# Supplementary material for: Evaluation of Diagnostic Potential of Epigenetically Deregulated MiRNAs in Epithelial Ovarian Cancer
Source: Front Oncol. 2021 Oct 7;11:681872. doi: 10.3389/fonc.2021.681872 (PMC8529058; doi:10.3389/fonc.2021.681872)
Supplement: Supplementary file 8 [file Table_4.docx]

Supplementary Table 4 : Represents target enrichment analysis of three candidate miRNA.

|  | | | | | | | |
| --- | --- | --- | --- | --- | --- | --- | --- |
| **Gene Symbol** | **p-value** | **FDR** | **Odd ratio** | **Number of interactions** | **microRNA 1** | **microRNA 2** | **microRNA 3** |
| FAM118B | 2.00E-05 | 0.045786 | 0.0318182 | 3 | hsa-miR-141-3p | hsa-miR-200c-3p | hsa-miR-205-5p |
| B3GNT2 | 3.72E-04 | 0.094769 | 0.0204545 | 2 | hsa-miR-141-3p | hsa-miR-200c-3p |  |
| DUSP7 | 2.08E-04 | 0.094769 | 0.0636364 | 3 | hsa-miR-141-3p | hsa-miR-200c-3p | hsa-miR-205-5p |
| KIAA1430 | 3.72E-04 | 0.094769 | 0.0204545 | 2 | hsa-miR-141-3p | hsa-miR-200c-3p |  |
| PAPLN | 2.08E-04 | 0.094769 | 0.0636364 | 3 | hsa-miR-141-3p | hsa-miR-200c-3p | hsa-miR-205-5p |
| PAQR5 | 9.43E-05 | 0.094769 | 0.0500000 | 3 | hsa-miR-141-3p | hsa-miR-200c-3p | hsa-miR-205-5p |
| PLCL1 | 2.60E-04 | 0.094769 | 0.0681818 | 3 | hsa-miR-141-3p | hsa-miR-200c-3p | hsa-miR-205-5p |
| SIAH1 | 1.26E-04 | 0.094769 | 0.0545455 | 3 | hsa-miR-141-3p | hsa-miR-200c-3p | hsa-miR-205-5p |
| TAF12 | 3.72E-04 | 0.094769 | 0.0204545 | 2 | hsa-miR-141-3p | hsa-miR-200c-3p |  |
| CBX1 | 7.43E-04 | 0.121473 | 0.0272727 | 2 | hsa-miR-141-3p | hsa-miR-205-5p |  |
| LRP1 | 7.43E-04 | 0.121473 | 0.0272727 | 2 | hsa-miR-200c-3p | hsa-miR-205-5p |  |
| MPRIP | 5.54E-04 | 0.121473 | 0.0863636 | 3 | hsa-miR-141-3p | hsa-miR-200c-3p | hsa-miR-205-5p |
| PHF16 | 7.43E-04 | 0.121473 | 0.0272727 | 2 | hsa-miR-200c-3p | hsa-miR-205-5p |  |
| TIAL1 | 6.51E-04 | 0.121473 | 0.0909091 | 3 | hsa-miR-141-3p | hsa-miR-200c-3p | hsa-miR-205-5p |
| CALU | 8.80E-04 | 0.125911 | 0.1000000 | 3 | hsa-miR-141-3p | hsa-miR-200c-3p | hsa-miR-205-5p |
| NDNF | 8.80E-04 | 0.125911 | 0.1000000 | 3 | hsa-miR-141-3p | hsa-miR-200c-3p | hsa-miR-205-5p |
| ELK3 | 1.01E-03 | 0.128709 | 0.1045455 | 3 | hsa-miR-141-3p | hsa-miR-200c-3p | hsa-miR-205-5p |
| SUGT1 | 1.01E-03 | 0.128709 | 0.1045455 | 3 | hsa-miR-141-3p | hsa-miR-200c-3p | hsa-miR-205-5p |
| ERRFI1 | 1.23E-03 | 0.148718 | 0.0340909 | 2 | hsa-miR-200c-3p | hsa-miR-205-5p |  |
| LYSMD3 | 1.31E-03 | 0.150439 | 0.1136364 | 3 | hsa-miR-141-3p | hsa-miR-200c-3p | hsa-miR-205-5p |
| GJC1 | 1.87E-03 | 0.171422 | 0.1272727 | 3 | hsa-miR-141-3p | hsa-miR-200c-3p | hsa-miR-205-5p |
| HSPA13 | 1.87E-03 | 0.171422 | 0.1272727 | 3 | hsa-miR-141-3p | hsa-miR-200c-3p | hsa-miR-205-5p |
| NPC1 | 1.85E-03 | 0.171422 | 0.0409091 | 2 | hsa-miR-141-3p | hsa-miR-200c-3p |  |
| SERINC1 | 1.85E-03 | 0.171422 | 0.0409091 | 2 | hsa-miR-141-3p | hsa-miR-200c-3p |  |
| SERPINC1 | 1.85E-03 | 0.171422 | 0.0409091 | 2 | hsa-miR-141-3p | hsa-miR-200c-3p |  |
| BAP1 | 2.58E-03 | 0.178704 | 0.0477273 | 2 | hsa-miR-141-3p | hsa-miR-200c-3p |  |
| CRMP1 | 2.58E-03 | 0.178704 | 0.0477273 | 2 | hsa-miR-141-3p | hsa-miR-205-5p |  |
| IDS | 2.58E-03 | 0.178704 | 0.0477273 | 2 | hsa-miR-141-3p | hsa-miR-200c-3p |  |
| LPAR1 | 2.58E-03 | 0.178704 | 0.0477273 | 2 | hsa-miR-200c-3p | hsa-miR-205-5p |  |
| PTCHD3 | 2.58E-03 | 0.178704 | 0.0477273 | 2 | hsa-miR-141-3p | hsa-miR-200c-3p |  |
| PTP4A1 | 2.57E-03 | 0.178704 | 0.1409091 | 3 | hsa-miR-141-3p | hsa-miR-200c-3p | hsa-miR-205-5p |
| SLC14A1 | 2.58E-03 | 0.178704 | 0.0477273 | 2 | hsa-miR-141-3p | hsa-miR-200c-3p |  |
| TP53INP1 | 2.57E-03 | 0.178704 | 0.1409091 | 3 | hsa-miR-141-3p | hsa-miR-200c-3p | hsa-miR-205-5p |
| AGPAT6 | 7.97E-03 | 0.189344 | 0.0818182 | 2 | hsa-miR-141-3p | hsa-miR-205-5p |  |
| ANO6 | 1.26E-02 | 0.189344 | 0.1022727 | 2 | hsa-miR-141-3p | hsa-miR-200c-3p |  |
| ARHGAP24 | 1.43E-02 | 0.189344 | 0.1090909 | 2 | hsa-miR-141-3p | hsa-miR-205-5p |  |
| ARL5A | 1.43E-02 | 0.189344 | 0.1090909 | 2 | hsa-miR-141-3p | hsa-miR-200c-3p |  |
| B3GNT5 | 4.39E-03 | 0.189344 | 0.0613636 | 2 | hsa-miR-141-3p | hsa-miR-205-5p |  |
| CADM1 | 1.26E-02 | 0.189344 | 0.2363636 | 3 | hsa-miR-141-3p | hsa-miR-200c-3p | hsa-miR-205-5p |
| CASC4 | 4.39E-03 | 0.189344 | 0.0613636 | 2 | hsa-miR-200c-3p | hsa-miR-205-5p |  |
| CDC42BPB | 3.42E-03 | 0.189344 | 0.0545455 | 2 | hsa-miR-141-3p | hsa-miR-205-5p |  |
| CDH7 | 1.09E-02 | 0.189344 | 0.0954545 | 2 | hsa-miR-200c-3p | hsa-miR-205-5p |  |
| CDK17 | 1.43E-02 | 0.189344 | 0.1090909 | 2 | hsa-miR-141-3p | hsa-miR-200c-3p |  |
| CFL2 | 1.05E-02 | 0.189344 | 0.2227273 | 3 | hsa-miR-141-3p | hsa-miR-200c-3p | hsa-miR-205-5p |
| CHRDL1 | 6.66E-03 | 0.189344 | 0.0750000 | 2 | hsa-miR-141-3p | hsa-miR-200c-3p |  |
| CLASP2 | 1.26E-02 | 0.189344 | 0.1022727 | 2 | hsa-miR-141-3p | hsa-miR-200c-3p |  |
| CLIP2 | 9.39E-03 | 0.189344 | 0.0886364 | 2 | hsa-miR-141-3p | hsa-miR-200c-3p |  |
| CNP | 4.39E-03 | 0.189344 | 0.0613636 | 2 | hsa-miR-141-3p | hsa-miR-205-5p |  |
| CSNK1G3 | 1.43E-02 | 0.189344 | 0.1090909 | 2 | hsa-miR-141-3p | hsa-miR-200c-3p |  |
| CTNND2 | 9.39E-03 | 0.189344 | 0.0886364 | 2 | hsa-miR-141-3p | hsa-miR-200c-3p |  |
| DCDC2 | 5.47E-03 | 0.189344 | 0.0681818 | 2 | hsa-miR-200c-3p | hsa-miR-205-5p |  |
| DZIP1 | 5.47E-03 | 0.189344 | 0.0681818 | 2 | hsa-miR-141-3p | hsa-miR-200c-3p |  |
| EGLN1 | 9.39E-03 | 0.189344 | 0.0886364 | 2 | hsa-miR-141-3p | hsa-miR-200c-3p |  |
| ELAVL4 | 3.12E-03 | 0.189344 | 0.1500000 | 3 | hsa-miR-141-3p | hsa-miR-200c-3p | hsa-miR-205-5p |
| ELMOD2 | 5.47E-03 | 0.189344 | 0.0681818 | 2 | hsa-miR-141-3p | hsa-miR-200c-3p |  |
| ESRRG | 7.57E-03 | 0.189344 | 0.2000000 | 3 | hsa-miR-141-3p | hsa-miR-200c-3p | hsa-miR-205-5p |
| FAM84B | 1.09E-02 | 0.189344 | 0.0954545 | 2 | hsa-miR-141-3p | hsa-miR-205-5p |  |
| FAM8A1 | 5.47E-03 | 0.189344 | 0.0681818 | 2 | hsa-miR-141-3p | hsa-miR-200c-3p |  |
| FBXO22 | 3.42E-03 | 0.189344 | 0.0545455 | 2 | hsa-miR-200c-3p | hsa-miR-205-5p |  |
| FBXW2 | 1.09E-02 | 0.189344 | 0.0954545 | 2 | hsa-miR-141-3p | hsa-miR-200c-3p |  |
| FLI1 | 1.26E-02 | 0.189344 | 0.1022727 | 2 | hsa-miR-141-3p | hsa-miR-200c-3p |  |
| FLJ20373 | 1.09E-02 | 0.189344 | 0.0954545 | 2 | hsa-miR-141-3p | hsa-miR-200c-3p |  |
| FOXF1 | 1.26E-02 | 0.189344 | 0.1022727 | 2 | hsa-miR-200c-3p | hsa-miR-205-5p |  |
| FRMD6 | 1.09E-02 | 0.189344 | 0.0954545 | 2 | hsa-miR-141-3p | hsa-miR-200c-3p |  |
| FSD1L | 1.26E-02 | 0.189344 | 0.1022727 | 2 | hsa-miR-141-3p | hsa-miR-205-5p |  |
| GAD2 | 1.26E-02 | 0.189344 | 0.1022727 | 2 | hsa-miR-141-3p | hsa-miR-200c-3p |  |
| GATA3 | 7.97E-03 | 0.189344 | 0.0818182 | 2 | hsa-miR-141-3p | hsa-miR-205-5p |  |
| GREB1L | 1.43E-02 | 0.189344 | 0.1090909 | 2 | hsa-miR-141-3p | hsa-miR-200c-3p |  |
| HCN1 | 7.05E-03 | 0.189344 | 0.1954545 | 3 | hsa-miR-141-3p | hsa-miR-200c-3p | hsa-miR-205-5p |
| HIPK3 | 8.11E-03 | 0.189344 | 0.2045455 | 3 | hsa-miR-141-3p | hsa-miR-200c-3p | hsa-miR-205-5p |
| HS3ST1 | 3.42E-03 | 0.189344 | 0.0545455 | 2 | hsa-miR-200c-3p | hsa-miR-205-5p |  |
| IQCJ-SCHIP1 | 9.39E-03 | 0.189344 | 0.0886364 | 2 | hsa-miR-141-3p | hsa-miR-200c-3p |  |
| KATNAL1 | 4.39E-03 | 0.189344 | 0.0613636 | 2 | hsa-miR-141-3p | hsa-miR-200c-3p |  |
| KLF12 | 1.12E-02 | 0.189344 | 0.2272727 | 3 | hsa-miR-141-3p | hsa-miR-200c-3p | hsa-miR-205-5p |
| KLF3 | 7.05E-03 | 0.189344 | 0.1954545 | 3 | hsa-miR-141-3p | hsa-miR-200c-3p | hsa-miR-205-5p |
| LPIN2 | 4.39E-03 | 0.189344 | 0.0613636 | 2 | hsa-miR-141-3p | hsa-miR-200c-3p |  |
| LRP4 | 1.26E-02 | 0.189344 | 0.1022727 | 2 | hsa-miR-200c-3p | hsa-miR-205-5p |  |
| LRRC8A | 6.66E-03 | 0.189344 | 0.0750000 | 2 | hsa-miR-141-3p | hsa-miR-200c-3p |  |
| LYPD6 | 1.09E-02 | 0.189344 | 0.0954545 | 2 | hsa-miR-141-3p | hsa-miR-205-5p |  |
| MAP4K4 | 1.43E-02 | 0.189344 | 0.1090909 | 2 | hsa-miR-141-3p | hsa-miR-200c-3p |  |
| MIER3 | 4.44E-03 | 0.189344 | 0.1681818 | 3 | hsa-miR-141-3p | hsa-miR-200c-3p | hsa-miR-205-5p |
| MMD | 6.66E-03 | 0.189344 | 0.0750000 | 2 | hsa-miR-200c-3p | hsa-miR-205-5p |  |
| NCAM1 | 6.66E-03 | 0.189344 | 0.0750000 | 2 | hsa-miR-141-3p | hsa-miR-200c-3p |  |
| NEK6 | 9.39E-03 | 0.189344 | 0.0886364 | 2 | hsa-miR-141-3p | hsa-miR-205-5p |  |
| NRCAM | 5.47E-03 | 0.189344 | 0.0681818 | 2 | hsa-miR-141-3p | hsa-miR-205-5p |  |
| NUP35 | 6.66E-03 | 0.189344 | 0.0750000 | 2 | hsa-miR-200c-3p | hsa-miR-205-5p |  |
| NXPH1 | 9.39E-03 | 0.189344 | 0.0886364 | 2 | hsa-miR-141-3p | hsa-miR-200c-3p |  |
| OSBPL11 | 3.42E-03 | 0.189344 | 0.0545455 | 2 | hsa-miR-141-3p | hsa-miR-200c-3p |  |
| P2RY1 | 7.97E-03 | 0.189344 | 0.0818182 | 2 | hsa-miR-141-3p | hsa-miR-205-5p |  |
| PHB2 | 1.26E-02 | 0.189344 | 0.1022727 | 2 | hsa-miR-141-3p | hsa-miR-200c-3p |  |
| PHYHIPL | 6.66E-03 | 0.189344 | 0.0750000 | 2 | hsa-miR-141-3p | hsa-miR-205-5p |  |
| PPP1R15B | 1.43E-02 | 0.189344 | 0.1090909 | 2 | hsa-miR-141-3p | hsa-miR-205-5p |  |
| PTEN | 1.42E-02 | 0.189344 | 0.2454545 | 3 | hsa-miR-141-3p | hsa-miR-200c-3p | hsa-miR-205-5p |
| RBFOX3 | 5.47E-03 | 0.189344 | 0.0681818 | 2 | hsa-miR-200c-3p | hsa-miR-205-5p |  |
| RBM47 | 1.43E-02 | 0.189344 | 0.1090909 | 2 | hsa-miR-141-3p | hsa-miR-205-5p |  |
| SCD5 | 4.39E-03 | 0.189344 | 0.0613636 | 2 | hsa-miR-141-3p | hsa-miR-205-5p |  |
| SCHIP1 | 9.39E-03 | 0.189344 | 0.0886364 | 2 | hsa-miR-141-3p | hsa-miR-200c-3p |  |
| SEC24A | 6.66E-03 | 0.189344 | 0.0750000 | 2 | hsa-miR-141-3p | hsa-miR-200c-3p |  |
| SECISBP2L | 1.26E-02 | 0.189344 | 0.1022727 | 2 | hsa-miR-200c-3p | hsa-miR-205-5p |  |
| SFXN1 | 5.47E-03 | 0.189344 | 0.0681818 | 2 | hsa-miR-141-3p | hsa-miR-200c-3p |  |
| SHROOM4 | 1.26E-02 | 0.189344 | 0.1022727 | 2 | hsa-miR-141-3p | hsa-miR-200c-3p |  |
| SLC5A3 | 4.08E-03 | 0.189344 | 0.1636364 | 3 | hsa-miR-141-3p | hsa-miR-200c-3p | hsa-miR-205-5p |
| SRSF10 | 1.42E-02 | 0.189344 | 0.2454545 | 3 | hsa-miR-141-3p | hsa-miR-200c-3p | hsa-miR-205-5p |
| SULF1 | 1.09E-02 | 0.189344 | 0.0954545 | 2 | hsa-miR-200c-3p | hsa-miR-205-5p |  |
| SYNJ2BP | 6.66E-03 | 0.189344 | 0.0750000 | 2 | hsa-miR-200c-3p | hsa-miR-205-5p |  |
| TAPT1 | 5.47E-03 | 0.189344 | 0.0681818 | 2 | hsa-miR-141-3p | hsa-miR-205-5p |  |
| TFAP2A | 9.39E-03 | 0.189344 | 0.0886364 | 2 | hsa-miR-141-3p | hsa-miR-200c-3p |  |
| TMEM237 | 6.66E-03 | 0.189344 | 0.0750000 | 2 | hsa-miR-141-3p | hsa-miR-200c-3p |  |
| WIPF1 | 6.66E-03 | 0.189344 | 0.0750000 | 2 | hsa-miR-141-3p | hsa-miR-200c-3p |  |
| WWC3 | 1.43E-02 | 0.189344 | 0.1090909 | 2 | hsa-miR-200c-3p | hsa-miR-205-5p |  |
| YAP1 | 4.39E-03 | 0.189344 | 0.0613636 | 2 | hsa-miR-141-3p | hsa-miR-205-5p |  |
| ZEB1 | 3.12E-03 | 0.189344 | 0.1500000 | 3 | hsa-miR-141-3p | hsa-miR-200c-3p | hsa-miR-205-5p |
| ZEB2 | 6.56E-03 | 0.189344 | 0.1909091 | 3 | hsa-miR-141-3p | hsa-miR-200c-3p | hsa-miR-205-5p |
| ZSWIM4 | 5.47E-03 | 0.189344 | 0.0681818 | 2 | hsa-miR-200c-3p | hsa-miR-205-5p |  |
| AKAP2 | 1.62E-02 | 0.193746 | 0.1159091 | 2 | hsa-miR-141-3p | hsa-miR-200c-3p |  |
| DOCK4 | 1.62E-02 | 0.193746 | 0.1159091 | 2 | hsa-miR-141-3p | hsa-miR-200c-3p |  |
| GPR63 | 1.62E-02 | 0.193746 | 0.1159091 | 2 | hsa-miR-141-3p | hsa-miR-200c-3p |  |
| IRF2BPL | 1.62E-02 | 0.193746 | 0.1159091 | 2 | hsa-miR-141-3p | hsa-miR-205-5p |  |
| LUC7L3 | 1.62E-02 | 0.193746 | 0.1159091 | 2 | hsa-miR-141-3p | hsa-miR-205-5p |  |
| MED13L | 1.62E-02 | 0.193746 | 0.1159091 | 2 | hsa-miR-141-3p | hsa-miR-205-5p |  |
| NKD1 | 1.62E-02 | 0.193746 | 0.1159091 | 2 | hsa-miR-200c-3p | hsa-miR-205-5p |  |
| PALM2-AKAP2 | 1.62E-02 | 0.193746 | 0.1159091 | 2 | hsa-miR-141-3p | hsa-miR-200c-3p |  |
| PDS5B | 1.62E-02 | 0.193746 | 0.1159091 | 2 | hsa-miR-141-3p | hsa-miR-200c-3p |  |
| PRKAR1A | 1.62E-02 | 0.193746 | 0.1159091 | 2 | hsa-miR-141-3p | hsa-miR-200c-3p |  |
| RANBP9 | 1.62E-02 | 0.193746 | 0.1159091 | 2 | hsa-miR-141-3p | hsa-miR-200c-3p |  |
| S100PBP | 1.62E-02 | 0.193746 | 0.1159091 | 2 | hsa-miR-141-3p | hsa-miR-200c-3p |  |
| SIX1 | 1.62E-02 | 0.193746 | 0.1159091 | 2 | hsa-miR-141-3p | hsa-miR-200c-3p |  |
| SLC38A2 | 1.62E-02 | 0.193746 | 0.1159091 | 2 | hsa-miR-141-3p | hsa-miR-200c-3p |  |
| SLK | 1.62E-02 | 0.193746 | 0.1159091 | 2 | hsa-miR-200c-3p | hsa-miR-205-5p |  |
| TBCEL | 1.62E-02 | 0.193746 | 0.1159091 | 2 | hsa-miR-200c-3p | hsa-miR-205-5p |  |
| TLN2 | 1.62E-02 | 0.193746 | 0.1159091 | 2 | hsa-miR-141-3p | hsa-miR-200c-3p |  |
| VTI1B | 1.62E-02 | 0.193746 | 0.1159091 | 2 | hsa-miR-200c-3p | hsa-miR-205-5p |  |
| ANK2 | 1.81E-02 | 0.208531 | 0.1227273 | 2 | hsa-miR-141-3p | hsa-miR-205-5p |  |
| DLC1 | 1.81E-02 | 0.208531 | 0.1227273 | 2 | hsa-miR-141-3p | hsa-miR-200c-3p |  |
| ETF1 | 1.81E-02 | 0.208531 | 0.1227273 | 2 | hsa-miR-200c-3p | hsa-miR-205-5p |  |
| PTCH1 | 1.81E-02 | 0.208531 | 0.1227273 | 2 | hsa-miR-141-3p | hsa-miR-200c-3p |  |
| SPAG9 | 1.81E-02 | 0.208531 | 0.1227273 | 2 | hsa-miR-141-3p | hsa-miR-200c-3p |  |
| STRN | 1.76E-02 | 0.208531 | 0.2636364 | 3 | hsa-miR-141-3p | hsa-miR-200c-3p | hsa-miR-205-5p |
| TMEM136 | 1.81E-02 | 0.208531 | 0.1227273 | 2 | hsa-miR-200c-3p | hsa-miR-205-5p |  |
| VEGFA | 1.81E-02 | 0.208531 | 0.1227273 | 2 | hsa-miR-200c-3p | hsa-miR-205-5p |  |
| ADD3 | 2.46E-02 | 0.212909 | 0.1431818 | 2 | hsa-miR-141-3p | hsa-miR-200c-3p |  |
| ATF7IP | 2.02E-02 | 0.212909 | 0.1295455 | 2 | hsa-miR-141-3p | hsa-miR-200c-3p |  |
| ATP2A2 | 2.24E-02 | 0.212909 | 0.1363636 | 2 | hsa-miR-141-3p | hsa-miR-200c-3p |  |
| BICC1 | 2.70E-02 | 0.212909 | 0.1500000 | 2 | hsa-miR-200c-3p | hsa-miR-205-5p |  |
| C6orf120 | 2.70E-02 | 0.212909 | 0.1500000 | 2 | hsa-miR-141-3p | hsa-miR-200c-3p |  |
| CACNA2D1 | 2.70E-02 | 0.212909 | 0.1500000 | 2 | hsa-miR-141-3p | hsa-miR-200c-3p |  |
| CDC27 | 2.70E-02 | 0.212909 | 0.1500000 | 2 | hsa-miR-200c-3p | hsa-miR-205-5p |  |
| CDC42EP3 | 2.46E-02 | 0.212909 | 0.1431818 | 2 | hsa-miR-141-3p | hsa-miR-200c-3p |  |
| DDX5 | 2.02E-02 | 0.212909 | 0.1295455 | 2 | hsa-miR-141-3p | hsa-miR-205-5p |  |
| DUSP1 | 2.02E-02 | 0.212909 | 0.1295455 | 2 | hsa-miR-141-3p | hsa-miR-200c-3p |  |
| ELF2 | 2.70E-02 | 0.212909 | 0.1500000 | 2 | hsa-miR-141-3p | hsa-miR-200c-3p |  |
| ERG | 2.02E-02 | 0.212909 | 0.1295455 | 2 | hsa-miR-141-3p | hsa-miR-200c-3p |  |
| FNBP1L | 2.02E-02 | 0.212909 | 0.1295455 | 2 | hsa-miR-141-3p | hsa-miR-205-5p |  |
| GIGYF1 | 2.46E-02 | 0.212909 | 0.1431818 | 2 | hsa-miR-141-3p | hsa-miR-200c-3p |  |
| GLCCI1 | 2.46E-02 | 0.212909 | 0.1431818 | 2 | hsa-miR-141-3p | hsa-miR-200c-3p |  |
| HDAC4 | 2.70E-02 | 0.212909 | 0.1500000 | 2 | hsa-miR-141-3p | hsa-miR-200c-3p |  |
| HMGB1 | 2.46E-02 | 0.212909 | 0.1431818 | 2 | hsa-miR-141-3p | hsa-miR-205-5p |  |
| KIF26B | 2.24E-02 | 0.212909 | 0.1363636 | 2 | hsa-miR-200c-3p | hsa-miR-205-5p |  |
| KLF6 | 2.24E-02 | 0.212909 | 0.1363636 | 2 | hsa-miR-141-3p | hsa-miR-200c-3p |  |
| KMT2C | 2.70E-02 | 0.212909 | 0.1500000 | 2 | hsa-miR-141-3p | hsa-miR-200c-3p |  |
| LRP1B | 2.70E-02 | 0.212909 | 0.1500000 | 2 | hsa-miR-141-3p | hsa-miR-200c-3p |  |
| MAGI2 | 2.70E-02 | 0.212909 | 0.1500000 | 2 | hsa-miR-141-3p | hsa-miR-205-5p |  |
| MBOAT2 | 2.46E-02 | 0.212909 | 0.1431818 | 2 | hsa-miR-141-3p | hsa-miR-200c-3p |  |
| MIB1 | 2.46E-02 | 0.212909 | 0.1431818 | 2 | hsa-miR-141-3p | hsa-miR-200c-3p |  |
| NCOA3 | 2.24E-02 | 0.212909 | 0.1363636 | 2 | hsa-miR-141-3p | hsa-miR-200c-3p |  |
| NFIA | 2.27E-02 | 0.212909 | 0.2863636 | 3 | hsa-miR-141-3p | hsa-miR-200c-3p | hsa-miR-205-5p |
| NPTX1 | 2.70E-02 | 0.212909 | 0.1500000 | 2 | hsa-miR-141-3p | hsa-miR-200c-3p |  |
| ONECUT1 | 2.46E-02 | 0.212909 | 0.1431818 | 2 | hsa-miR-141-3p | hsa-miR-205-5p |  |
| PPP2CA | 2.70E-02 | 0.212909 | 0.1500000 | 2 | hsa-miR-141-3p | hsa-miR-200c-3p |  |
| PPP4R2 | 2.24E-02 | 0.212909 | 0.1363636 | 2 | hsa-miR-141-3p | hsa-miR-200c-3p |  |
| PRKACB | 2.46E-02 | 0.212909 | 0.1431818 | 2 | hsa-miR-141-3p | hsa-miR-200c-3p |  |
| QKI | 2.16E-02 | 0.212909 | 0.2818182 | 3 | hsa-miR-141-3p | hsa-miR-200c-3p | hsa-miR-205-5p |
| RBM20 | 2.24E-02 | 0.212909 | 0.1363636 | 2 | hsa-miR-141-3p | hsa-miR-200c-3p |  |
| SPOPL | 2.24E-02 | 0.212909 | 0.1363636 | 2 | hsa-miR-141-3p | hsa-miR-205-5p |  |
| TET1 | 2.24E-02 | 0.212909 | 0.1363636 | 2 | hsa-miR-141-3p | hsa-miR-205-5p |  |
| THSD7A | 2.46E-02 | 0.212909 | 0.1431818 | 2 | hsa-miR-141-3p | hsa-miR-200c-3p |  |
| TRAPPC8 | 2.46E-02 | 0.212909 | 0.1431818 | 2 | hsa-miR-141-3p | hsa-miR-200c-3p |  |
| TSC22D1 | 2.24E-02 | 0.212909 | 0.1363636 | 2 | hsa-miR-200c-3p | hsa-miR-205-5p |  |
| TSHZ3 | 2.24E-02 | 0.212909 | 0.1363636 | 2 | hsa-miR-141-3p | hsa-miR-205-5p |  |
| UBA6 | 2.46E-02 | 0.212909 | 0.1431818 | 2 | hsa-miR-141-3p | hsa-miR-200c-3p |  |
| WDFY3 | 2.24E-02 | 0.212909 | 0.1363636 | 2 | hsa-miR-141-3p | hsa-miR-200c-3p |  |
| ZBTB38 | 2.24E-02 | 0.212909 | 0.1363636 | 2 | hsa-miR-200c-3p | hsa-miR-205-5p |  |
| ZNF436 | 2.02E-02 | 0.212909 | 0.1295455 | 2 | hsa-miR-141-3p | hsa-miR-205-5p |  |
| CERS6 | 2.95E-02 | 0.223567 | 0.1568182 | 2 | hsa-miR-141-3p | hsa-miR-200c-3p |  |
| DMXL1 | 2.95E-02 | 0.223567 | 0.1568182 | 2 | hsa-miR-141-3p | hsa-miR-205-5p |  |
| ITSN1 | 2.95E-02 | 0.223567 | 0.1568182 | 2 | hsa-miR-141-3p | hsa-miR-200c-3p |  |
| NKAP | 2.95E-02 | 0.223567 | 0.1568182 | 2 | hsa-miR-200c-3p | hsa-miR-205-5p |  |
| NRP2 | 2.95E-02 | 0.223567 | 0.1568182 | 2 | hsa-miR-141-3p | hsa-miR-200c-3p |  |
| PCDH8 | 2.95E-02 | 0.223567 | 0.1568182 | 2 | hsa-miR-141-3p | hsa-miR-200c-3p |  |
| RASA2 | 2.95E-02 | 0.223567 | 0.1568182 | 2 | hsa-miR-141-3p | hsa-miR-200c-3p |  |
| TRAM2 | 2.95E-02 | 0.223567 | 0.1568182 | 2 | hsa-miR-141-3p | hsa-miR-205-5p |  |
| TTLL7 | 2.95E-02 | 0.223567 | 0.1568182 | 2 | hsa-miR-141-3p | hsa-miR-205-5p |  |
| ZNF638 | 2.95E-02 | 0.223567 | 0.1568182 | 2 | hsa-miR-141-3p | hsa-miR-205-5p |  |
| ANKRD50 | 3.21E-02 | 0.236059 | 0.1636364 | 2 | hsa-miR-141-3p | hsa-miR-205-5p |  |
| BRMS1L | 3.21E-02 | 0.236059 | 0.1636364 | 2 | hsa-miR-141-3p | hsa-miR-200c-3p |  |
| CGGBP1 | 3.21E-02 | 0.236059 | 0.1636364 | 2 | hsa-miR-141-3p | hsa-miR-200c-3p |  |
| PPP3R1 | 3.21E-02 | 0.236059 | 0.1636364 | 2 | hsa-miR-141-3p | hsa-miR-205-5p |  |
| PRKCE | 3.21E-02 | 0.236059 | 0.1636364 | 2 | hsa-miR-141-3p | hsa-miR-205-5p |  |
| ROCK2 | 3.21E-02 | 0.236059 | 0.1636364 | 2 | hsa-miR-200c-3p | hsa-miR-205-5p |  |
| SNX27 | 3.21E-02 | 0.236059 | 0.1636364 | 2 | hsa-miR-141-3p | hsa-miR-205-5p |  |
| TRHDE | 3.21E-02 | 0.236059 | 0.1636364 | 2 | hsa-miR-141-3p | hsa-miR-200c-3p |  |
| WAPAL | 3.21E-02 | 0.236059 | 0.1636364 | 2 | hsa-miR-141-3p | hsa-miR-200c-3p |  |
| ATAD2B | 3.75E-02 | 0.237411 | 0.1772727 | 2 | hsa-miR-141-3p | hsa-miR-200c-3p |  |
| CAMSAP2 | 3.47E-02 | 0.237411 | 0.1704545 | 2 | hsa-miR-141-3p | hsa-miR-200c-3p |  |
| CASD1 | 4.04E-02 | 0.237411 | 0.1840909 | 2 | hsa-miR-200c-3p | hsa-miR-205-5p |  |
| CDH11 | 3.47E-02 | 0.237411 | 0.1704545 | 2 | hsa-miR-200c-3p | hsa-miR-205-5p |  |
| CDK19 | 4.04E-02 | 0.237411 | 0.1840909 | 2 | hsa-miR-200c-3p | hsa-miR-205-5p |  |
| CHD2 | 4.04E-02 | 0.237411 | 0.1840909 | 2 | hsa-miR-141-3p | hsa-miR-200c-3p |  |
| CORO1C | 4.04E-02 | 0.237411 | 0.1840909 | 2 | hsa-miR-141-3p | hsa-miR-200c-3p |  |
| CSNK2A1 | 4.04E-02 | 0.237411 | 0.1840909 | 2 | hsa-miR-141-3p | hsa-miR-205-5p |  |
| ELL2 | 3.47E-02 | 0.237411 | 0.1704545 | 2 | hsa-miR-141-3p | hsa-miR-200c-3p |  |
| FRMD4A | 4.04E-02 | 0.237411 | 0.1840909 | 2 | hsa-miR-141-3p | hsa-miR-200c-3p |  |
| GATSL2 | 3.75E-02 | 0.237411 | 0.1772727 | 2 | hsa-miR-141-3p | hsa-miR-200c-3p |  |
| GPC6 | 4.04E-02 | 0.237411 | 0.1840909 | 2 | hsa-miR-141-3p | hsa-miR-200c-3p |  |
| HNRNPK | 3.75E-02 | 0.237411 | 0.1772727 | 2 | hsa-miR-200c-3p | hsa-miR-205-5p |  |
| HNRNPR | 3.47E-02 | 0.237411 | 0.1704545 | 2 | hsa-miR-141-3p | hsa-miR-205-5p |  |
| HS2ST1 | 3.75E-02 | 0.237411 | 0.1772727 | 2 | hsa-miR-141-3p | hsa-miR-200c-3p |  |
| INHBA | 3.47E-02 | 0.237411 | 0.1704545 | 2 | hsa-miR-200c-3p | hsa-miR-205-5p |  |
| MARCH6 | 3.47E-02 | 0.237411 | 0.1704545 | 2 | hsa-miR-141-3p | hsa-miR-200c-3p |  |
| MTF2 | 3.75E-02 | 0.237411 | 0.1772727 | 2 | hsa-miR-141-3p | hsa-miR-200c-3p |  |
| NR3C1 | 3.47E-02 | 0.237411 | 0.1704545 | 2 | hsa-miR-141-3p | hsa-miR-200c-3p |  |
| PARD6B | 4.04E-02 | 0.237411 | 0.1840909 | 2 | hsa-miR-200c-3p | hsa-miR-205-5p |  |
| PCNX | 3.47E-02 | 0.237411 | 0.1704545 | 2 | hsa-miR-200c-3p | hsa-miR-205-5p |  |
| PPP2R5E | 3.47E-02 | 0.237411 | 0.1704545 | 2 | hsa-miR-141-3p | hsa-miR-200c-3p |  |
| PRKCA | 3.75E-02 | 0.237411 | 0.1772727 | 2 | hsa-miR-200c-3p | hsa-miR-205-5p |  |
| RAPGEF2 | 4.04E-02 | 0.237411 | 0.1840909 | 2 | hsa-miR-141-3p | hsa-miR-200c-3p |  |
| SOGA3 | 3.75E-02 | 0.237411 | 0.1772727 | 2 | hsa-miR-141-3p | hsa-miR-205-5p |  |
| SP4 | 4.04E-02 | 0.237411 | 0.1840909 | 2 | hsa-miR-141-3p | hsa-miR-205-5p |  |
| TXLNG | 4.04E-02 | 0.237411 | 0.1840909 | 2 | hsa-miR-141-3p | hsa-miR-200c-3p |  |
| YWHAG | 3.47E-02 | 0.237411 | 0.1704545 | 2 | hsa-miR-141-3p | hsa-miR-200c-3p |  |
| ACVR2A | 5.28E-02 | 0.246900 | 0.2113636 | 2 | hsa-miR-141-3p | hsa-miR-200c-3p |  |
| AFF1 | 4.95E-02 | 0.246900 | 0.2045455 | 2 | hsa-miR-141-3p | hsa-miR-200c-3p |  |
| APBB2 | 4.95E-02 | 0.246900 | 0.2045455 | 2 | hsa-miR-141-3p | hsa-miR-205-5p |  |
| ARID5B | 4.33E-02 | 0.246900 | 0.1909091 | 2 | hsa-miR-141-3p | hsa-miR-200c-3p |  |
| ASXL3 | 4.33E-02 | 0.246900 | 0.1909091 | 2 | hsa-miR-141-3p | hsa-miR-200c-3p |  |
| C16orf52 | 4.64E-02 | 0.246900 | 0.1977273 | 2 | hsa-miR-200c-3p | hsa-miR-205-5p |  |
| CCDC85C | 4.64E-02 | 0.246900 | 0.1977273 | 2 | hsa-miR-141-3p | hsa-miR-205-5p |  |
| CHD9 | 4.33E-02 | 0.246900 | 0.1909091 | 2 | hsa-miR-141-3p | hsa-miR-200c-3p |  |
| CTBP2 | 4.95E-02 | 0.246900 | 0.2045455 | 2 | hsa-miR-141-3p | hsa-miR-200c-3p |  |
| DR1 | 5.28E-02 | 0.246900 | 0.2113636 | 2 | hsa-miR-141-3p | hsa-miR-200c-3p |  |
| FAM46C | 4.64E-02 | 0.246900 | 0.1977273 | 2 | hsa-miR-141-3p | hsa-miR-200c-3p |  |
| GDF6 | 4.33E-02 | 0.246900 | 0.1909091 | 2 | hsa-miR-141-3p | hsa-miR-200c-3p |  |
| GNA13 | 4.64E-02 | 0.246900 | 0.1977273 | 2 | hsa-miR-141-3p | hsa-miR-200c-3p |  |
| HIPK1 | 4.33E-02 | 0.246900 | 0.1909091 | 2 | hsa-miR-141-3p | hsa-miR-200c-3p |  |
| LAMC1 | 4.64E-02 | 0.246900 | 0.1977273 | 2 | hsa-miR-200c-3p | hsa-miR-205-5p |  |
| MATR3 | 4.64E-02 | 0.246900 | 0.1977273 | 2 | hsa-miR-141-3p | hsa-miR-200c-3p |  |
| MGA | 4.33E-02 | 0.246900 | 0.1909091 | 2 | hsa-miR-200c-3p | hsa-miR-205-5p |  |
| NFASC | 4.64E-02 | 0.246900 | 0.1977273 | 2 | hsa-miR-141-3p | hsa-miR-200c-3p |  |
| NR2C2 | 4.95E-02 | 0.246900 | 0.2045455 | 2 | hsa-miR-141-3p | hsa-miR-200c-3p |  |
| PEAK1 | 4.33E-02 | 0.246900 | 0.1909091 | 2 | hsa-miR-141-3p | hsa-miR-200c-3p |  |
| PLXNA4 | 4.33E-02 | 0.246900 | 0.1909091 | 2 | hsa-miR-141-3p | hsa-miR-200c-3p |  |
| RALGPS2 | 4.95E-02 | 0.246900 | 0.2045455 | 2 | hsa-miR-141-3p | hsa-miR-200c-3p |  |
| RAP2C | 5.28E-02 | 0.246900 | 0.2113636 | 2 | hsa-miR-141-3p | hsa-miR-200c-3p |  |
| RFX7 | 4.33E-02 | 0.246900 | 0.1909091 | 2 | hsa-miR-141-3p | hsa-miR-200c-3p |  |
| RUNX1 | 4.33E-02 | 0.246900 | 0.1909091 | 2 | hsa-miR-141-3p | hsa-miR-205-5p |  |
| SETD9 | 4.95E-02 | 0.246900 | 0.2045455 | 2 | hsa-miR-141-3p | hsa-miR-200c-3p |  |
| SMARCD1 | 4.95E-02 | 0.246900 | 0.2045455 | 2 | hsa-miR-200c-3p | hsa-miR-205-5p |  |
| SNAP25 | 4.64E-02 | 0.246900 | 0.1977273 | 2 | hsa-miR-141-3p | hsa-miR-200c-3p |  |
| SRGAP1 | 4.95E-02 | 0.246900 | 0.2045455 | 2 | hsa-miR-200c-3p | hsa-miR-205-5p |  |
| ULK2 | 4.95E-02 | 0.246900 | 0.2045455 | 2 | hsa-miR-141-3p | hsa-miR-200c-3p |  |
| VPS13B | 4.95E-02 | 0.246900 | 0.2045455 | 2 | hsa-miR-141-3p | hsa-miR-205-5p |  |
| ZCCHC24 | 4.95E-02 | 0.246900 | 0.2045455 | 2 | hsa-miR-141-3p | hsa-miR-200c-3p |  |
| ZNF281 | 5.28E-02 | 0.246900 | 0.2113636 | 2 | hsa-miR-141-3p | hsa-miR-200c-3p |  |
| ETNK1 | 5.61E-02 | 0.250923 | 0.2181818 | 2 | hsa-miR-141-3p | hsa-miR-205-5p |  |
| FAM168B | 5.61E-02 | 0.250923 | 0.2181818 | 2 | hsa-miR-141-3p | hsa-miR-200c-3p |  |
| FNDC3B | 5.61E-02 | 0.250923 | 0.2181818 | 2 | hsa-miR-141-3p | hsa-miR-200c-3p |  |
| GAB1 | 5.61E-02 | 0.250923 | 0.2181818 | 2 | hsa-miR-141-3p | hsa-miR-200c-3p |  |
| MYO9A | 5.61E-02 | 0.250923 | 0.2181818 | 2 | hsa-miR-141-3p | hsa-miR-200c-3p |  |
| PAPOLG | 5.61E-02 | 0.250923 | 0.2181818 | 2 | hsa-miR-141-3p | hsa-miR-200c-3p |  |
| PLCXD3 | 5.61E-02 | 0.250923 | 0.2181818 | 2 | hsa-miR-141-3p | hsa-miR-200c-3p |  |
| RASSF8 | 5.61E-02 | 0.250923 | 0.2181818 | 2 | hsa-miR-141-3p | hsa-miR-200c-3p |  |
| SDC2 | 5.61E-02 | 0.250923 | 0.2181818 | 2 | hsa-miR-141-3p | hsa-miR-200c-3p |  |
| SLC23A2 | 5.61E-02 | 0.250923 | 0.2181818 | 2 | hsa-miR-141-3p | hsa-miR-200c-3p |  |
| TNRC6C | 5.61E-02 | 0.250923 | 0.2181818 | 2 | hsa-miR-141-3p | hsa-miR-205-5p |  |
| ZNF516 | 5.61E-02 | 0.250923 | 0.2181818 | 2 | hsa-miR-141-3p | hsa-miR-200c-3p |  |
| ZNF609 | 5.61E-02 | 0.250923 | 0.2181818 | 2 | hsa-miR-141-3p | hsa-miR-205-5p |  |
| AFF3 | 6.66E-02 | 0.253385 | 0.2386364 | 2 | hsa-miR-200c-3p | hsa-miR-205-5p |  |
| BEND4 | 6.66E-02 | 0.253385 | 0.2386364 | 2 | hsa-miR-141-3p | hsa-miR-205-5p |  |
| BRWD1 | 6.66E-02 | 0.253385 | 0.2386364 | 2 | hsa-miR-141-3p | hsa-miR-200c-3p |  |
| CCNJ | 6.30E-02 | 0.253385 | 0.2318182 | 2 | hsa-miR-200c-3p | hsa-miR-205-5p |  |
| CDK13 | 5.95E-02 | 0.253385 | 0.2250000 | 2 | hsa-miR-141-3p | hsa-miR-200c-3p |  |
| HLF | 5.95E-02 | 0.253385 | 0.2250000 | 2 | hsa-miR-141-3p | hsa-miR-200c-3p |  |
| KCND3 | 6.66E-02 | 0.253385 | 0.2386364 | 2 | hsa-miR-200c-3p | hsa-miR-205-5p |  |
| LSAMP | 5.95E-02 | 0.253385 | 0.2250000 | 2 | hsa-miR-141-3p | hsa-miR-205-5p |  |
| MED1 | 6.30E-02 | 0.253385 | 0.2318182 | 2 | hsa-miR-200c-3p | hsa-miR-205-5p |  |
| NACC2 | 5.95E-02 | 0.253385 | 0.2250000 | 2 | hsa-miR-200c-3p | hsa-miR-205-5p |  |
| NCOA2 | 6.30E-02 | 0.253385 | 0.2318182 | 2 | hsa-miR-141-3p | hsa-miR-200c-3p |  |
| NR6A1 | 6.66E-02 | 0.253385 | 0.2386364 | 2 | hsa-miR-141-3p | hsa-miR-205-5p |  |
| PHF21A | 6.30E-02 | 0.253385 | 0.2318182 | 2 | hsa-miR-141-3p | hsa-miR-200c-3p |  |
| PTPRG | 6.66E-02 | 0.253385 | 0.2386364 | 2 | hsa-miR-141-3p | hsa-miR-205-5p |  |
| SAMD8 | 6.66E-02 | 0.253385 | 0.2386364 | 2 | hsa-miR-141-3p | hsa-miR-200c-3p |  |
| TMEM170B | 6.66E-02 | 0.253385 | 0.2386364 | 2 | hsa-miR-141-3p | hsa-miR-200c-3p |  |
| ZFAND5 | 6.30E-02 | 0.253385 | 0.2318182 | 2 | hsa-miR-141-3p | hsa-miR-200c-3p |  |
| BBX | 7.03E-02 | 0.260472 | 0.2454545 | 2 | hsa-miR-141-3p | hsa-miR-200c-3p |  |
| C20orf194 | 7.03E-02 | 0.260472 | 0.2454545 | 2 | hsa-miR-141-3p | hsa-miR-205-5p |  |
| E2F3 | 7.03E-02 | 0.260472 | 0.2454545 | 2 | hsa-miR-141-3p | hsa-miR-200c-3p |  |
| EIF4E | 7.41E-02 | 0.260472 | 0.2522727 | 2 | hsa-miR-200c-3p | hsa-miR-205-5p |  |
| MARCKS | 7.41E-02 | 0.260472 | 0.2522727 | 2 | hsa-miR-200c-3p | hsa-miR-205-5p |  |
| MXI1 | 7.41E-02 | 0.260472 | 0.2522727 | 2 | hsa-miR-200c-3p | hsa-miR-205-5p |  |
| RNF11 | 7.03E-02 | 0.260472 | 0.2454545 | 2 | hsa-miR-141-3p | hsa-miR-200c-3p |  |
| RNF38 | 7.03E-02 | 0.260472 | 0.2454545 | 2 | hsa-miR-141-3p | hsa-miR-200c-3p |  |
| SLC4A4 | 7.03E-02 | 0.260472 | 0.2454545 | 2 | hsa-miR-200c-3p | hsa-miR-205-5p |  |
| SOX5 | 7.41E-02 | 0.260472 | 0.2522727 | 2 | hsa-miR-141-3p | hsa-miR-200c-3p |  |
| UBE2G1 | 7.03E-02 | 0.260472 | 0.2454545 | 2 | hsa-miR-141-3p | hsa-miR-205-5p |  |
| ZBTB16 | 7.41E-02 | 0.260472 | 0.2522727 | 2 | hsa-miR-141-3p | hsa-miR-200c-3p |  |
| AFF4 | 8.99E-02 | 0.261560 | 0.2795455 | 2 | hsa-miR-200c-3p | hsa-miR-205-5p |  |
| AGFG1 | 8.99E-02 | 0.261560 | 0.2795455 | 2 | hsa-miR-141-3p | hsa-miR-200c-3p |  |
| BACH2 | 8.58E-02 | 0.261560 | 0.2727273 | 2 | hsa-miR-141-3p | hsa-miR-200c-3p |  |
| CELF1 | 8.58E-02 | 0.261560 | 0.2727273 | 2 | hsa-miR-141-3p | hsa-miR-200c-3p |  |
| CSNK1A1 | 8.18E-02 | 0.261560 | 0.2659091 | 2 | hsa-miR-141-3p | hsa-miR-205-5p |  |
| FAM168A | 8.99E-02 | 0.261560 | 0.2795455 | 2 | hsa-miR-141-3p | hsa-miR-205-5p |  |
| GMFB | 8.18E-02 | 0.261560 | 0.2659091 | 2 | hsa-miR-200c-3p | hsa-miR-205-5p |  |
| HIC2 | 8.58E-02 | 0.261560 | 0.2727273 | 2 | hsa-miR-141-3p | hsa-miR-200c-3p |  |
| IL6ST | 8.18E-02 | 0.261560 | 0.2659091 | 2 | hsa-miR-200c-3p | hsa-miR-205-5p |  |
| KMT2A | 8.18E-02 | 0.261560 | 0.2659091 | 2 | hsa-miR-141-3p | hsa-miR-205-5p |  |
| MBD5 | 8.58E-02 | 0.261560 | 0.2727273 | 2 | hsa-miR-141-3p | hsa-miR-200c-3p |  |
| MBNL3 | 8.18E-02 | 0.261560 | 0.2659091 | 2 | hsa-miR-141-3p | hsa-miR-200c-3p |  |
| NIPBL | 8.58E-02 | 0.261560 | 0.2727273 | 2 | hsa-miR-141-3p | hsa-miR-200c-3p |  |
| PRDM16 | 8.99E-02 | 0.261560 | 0.2795455 | 2 | hsa-miR-200c-3p | hsa-miR-205-5p |  |
| PROX1 | 8.99E-02 | 0.261560 | 0.2795455 | 2 | hsa-miR-200c-3p | hsa-miR-205-5p |  |
| PTPRD | 8.58E-02 | 0.261560 | 0.2727273 | 2 | hsa-miR-141-3p | hsa-miR-205-5p |  |
| RBMS1 | 8.18E-02 | 0.261560 | 0.2659091 | 2 | hsa-miR-141-3p | hsa-miR-205-5p |  |
| CBL | 9.41E-02 | 0.263132 | 0.2863636 | 2 | hsa-miR-141-3p | hsa-miR-200c-3p |  |
| DCUN1D3 | 9.41E-02 | 0.263132 | 0.2863636 | 2 | hsa-miR-141-3p | hsa-miR-200c-3p |  |
| FAM63B | 9.41E-02 | 0.263132 | 0.2863636 | 2 | hsa-miR-200c-3p | hsa-miR-205-5p |  |
| GXYLT1 | 9.41E-02 | 0.263132 | 0.2863636 | 2 | hsa-miR-200c-3p | hsa-miR-205-5p |  |
| PAPD5 | 9.41E-02 | 0.263132 | 0.2863636 | 2 | hsa-miR-200c-3p | hsa-miR-205-5p |  |
| THRB | 9.41E-02 | 0.263132 | 0.2863636 | 2 | hsa-miR-141-3p | hsa-miR-200c-3p |  |
| AEBP2 | 9.84E-02 | 0.263802 | 0.2931818 | 2 | hsa-miR-200c-3p | hsa-miR-205-5p |  |
| FOXP1 | 1.03E-01 | 0.263802 | 0.3000000 | 2 | hsa-miR-141-3p | hsa-miR-200c-3p |  |
| MSL2 | 9.84E-02 | 0.263802 | 0.2931818 | 2 | hsa-miR-200c-3p | hsa-miR-205-5p |  |
| PLXNC1 | 1.03E-01 | 0.263802 | 0.3000000 | 2 | hsa-miR-141-3p | hsa-miR-200c-3p |  |
| SIK2 | 9.84E-02 | 0.263802 | 0.2931818 | 2 | hsa-miR-200c-3p | hsa-miR-205-5p |  |
| SYNCRIP | 1.03E-01 | 0.263802 | 0.3000000 | 2 | hsa-miR-141-3p | hsa-miR-200c-3p |  |
| XIAP | 9.84E-02 | 0.263802 | 0.2931818 | 2 | hsa-miR-141-3p | hsa-miR-200c-3p |  |
| ZCCHC14 | 9.84E-02 | 0.263802 | 0.2931818 | 2 | hsa-miR-200c-3p | hsa-miR-205-5p |  |
| ZNF148 | 9.84E-02 | 0.263802 | 0.2931818 | 2 | hsa-miR-200c-3p | hsa-miR-205-5p |  |
| BAZ2B | 1.12E-01 | 0.266099 | 0.3136364 | 2 | hsa-miR-141-3p | hsa-miR-200c-3p |  |
| CCNT2 | 1.16E-01 | 0.266099 | 0.3204545 | 2 | hsa-miR-141-3p | hsa-miR-200c-3p |  |
| ELAVL2 | 1.16E-01 | 0.266099 | 0.3204545 | 2 | hsa-miR-141-3p | hsa-miR-200c-3p |  |
| KCNB1 | 1.16E-01 | 0.266099 | 0.3204545 | 2 | hsa-miR-200c-3p | hsa-miR-205-5p |  |
| MBNL1 | 1.07E-01 | 0.266099 | 0.3068182 | 2 | hsa-miR-141-3p | hsa-miR-200c-3p |  |
| TEAD1 | 1.16E-01 | 0.266099 | 0.3204545 | 2 | hsa-miR-200c-3p | hsa-miR-205-5p |  |
| TNPO1 | 1.16E-01 | 0.266099 | 0.3204545 | 2 | hsa-miR-141-3p | hsa-miR-205-5p |  |
| ZBTB20 | 1.07E-01 | 0.266099 | 0.4772727 | 3 | hsa-miR-141-3p | hsa-miR-200c-3p | hsa-miR-205-5p |
| CLOCK | 1.21E-01 | 0.271277 | 0.3272727 | 2 | hsa-miR-141-3p | hsa-miR-200c-3p |  |
| ATXN1 | 1.30E-01 | 0.274105 | 0.3409091 | 2 | hsa-miR-141-3p | hsa-miR-200c-3p |  |
| MMP16 | 1.30E-01 | 0.274105 | 0.3409091 | 2 | hsa-miR-141-3p | hsa-miR-200c-3p |  |
| CREBRF | 1.40E-01 | 0.279651 | 0.3545455 | 2 | hsa-miR-141-3p | hsa-miR-205-5p |  |
| DYRK2 | 1.35E-01 | 0.279651 | 0.3477273 | 2 | hsa-miR-141-3p | hsa-miR-200c-3p |  |
| ERBB4 | 1.40E-01 | 0.279651 | 0.3545455 | 2 | hsa-miR-200c-3p | hsa-miR-205-5p |  |
| RPS6KA3 | 1.35E-01 | 0.279651 | 0.3477273 | 2 | hsa-miR-200c-3p | hsa-miR-205-5p |  |
| TCF4 | 1.45E-01 | 0.282529 | 0.3613636 | 2 | hsa-miR-141-3p | hsa-miR-200c-3p |  |
| CAND1 | 1.55E-01 | 0.286099 | 0.3750000 | 2 | hsa-miR-200c-3p | hsa-miR-205-5p |  |
| SOX6 | 1.55E-01 | 0.286099 | 0.3750000 | 2 | hsa-miR-141-3p | hsa-miR-200c-3p |  |
| RORA | 1.60E-01 | 0.287488 | 0.3818182 | 2 | hsa-miR-141-3p | hsa-miR-205-5p |  |
| ABI2 | 1.76E-01 | 0.290753 | 0.4022727 | 2 | hsa-miR-200c-3p | hsa-miR-205-5p |  |
| HIPK2 | 1.71E-01 | 0.290753 | 0.3954545 | 2 | hsa-miR-141-3p | hsa-miR-200c-3p |  |
| TNRC6B | 1.71E-01 | 0.290753 | 0.3954545 | 2 | hsa-miR-141-3p | hsa-miR-205-5p |  |
| BNC2 | 1.81E-01 | 0.292834 | 0.4090909 | 2 | hsa-miR-141-3p | hsa-miR-200c-3p |  |
| CREB1 | 1.81E-01 | 0.292834 | 0.4090909 | 2 | hsa-miR-200c-3p | hsa-miR-205-5p |  |
| ZNF652 | 1.87E-01 | 0.296462 | 0.4159091 | 2 | hsa-miR-200c-3p | hsa-miR-205-5p |  |
| CBX5 | 1.92E-01 | 0.297117 | 0.4227273 | 2 | hsa-miR-141-3p | hsa-miR-200c-3p |  |
| AAK1 | 2.21E-01 | 0.312403 | 0.4568182 | 2 | hsa-miR-200c-3p | hsa-miR-205-5p |  |
| LCOR | 2.26E-01 | 0.312403 | 0.4636364 | 2 | hsa-miR-200c-3p | hsa-miR-205-5p |  |
| TAOK1 | 2.21E-01 | 0.312403 | 0.4568182 | 2 | hsa-miR-200c-3p | hsa-miR-205-5p |  |
| NFIB | 2.68E-01 | 0.334840 | 0.5113636 | 2 | hsa-miR-200c-3p | hsa-miR-205-5p |  |
| NUFIP2 | 2.68E-01 | 0.334840 | 0.5113636 | 2 | hsa-miR-141-3p | hsa-miR-200c-3p |  |
