# Supplementary material for: Evaluation of Diagnostic Potential of Epigenetically Deregulated MiRNAs in Epithelial Ovarian Cancer
Source: Front Oncol. 2021 Oct 7;11:681872. doi: 10.3389/fonc.2021.681872 (PMC8529058; doi:10.3389/fonc.2021.681872)
Supplement: Supplementary file 9 [file Table_5.docx]

| KEGG ID | miRNAs | Term name | Relevance to cancer | Reference |
| --- | --- | --- | --- | --- |
| hsa05200 | hsa-miR-200c, miR-141, miR-205, let-7, miR-181, miR-155, miR-10b, miR-520 | Pathways in cancer | - | - |
| hsa04010 | hsa-miR205, miR-200c, miR-141, miR-450b-5p | MAPK signaling pathway | MAPK pathway play important role such as regulation of cell growth, proliferation and apoptosis in cancer cells. | (26,27) |
| hsa04310 | hsa-miR-141, miR-200 family | Wnt signaling pathway | Wnt signaling pathway regulated many physiological process including cell migration, polarization, and EMT. | (28–31) |
| hsa04150 | hsa-miR1911, has-miR-205, has-miR-141 | mTOR signaling pathway | Mammalian target of rapamycin (mTOR) signaling pathway regulates cell proliferation, growth and survival of the cell in cancer | (10,32–38) |
| hsa04390 | has-miR-181a, has-miR-205, has-miR-200 | Hippo signaling pathway | Hippo signaling pathway induces proliferation, invasion, chemoresistance and metastasis in cancer. | (39–41) |
| hsa04015 | miRNA-141/200c cluster | Rap1 signaling pathway | Rap1 signaling pathway indirectly involve in regulation of cancer cell metastases and invasion during carcinogenesis | (42,43) |
| hsa05206 | hsa-miR-205, has-miR-141, has-miR-200 family, has- | MicroRNAs in cancer | - | (16,17,44) |

Supplementary Table 5. Recent research papers elucidating the relevance of significantly enriched KEGG terms in cancer. The most relevant cancer-associated-functions of these pathway are enlisted in this table.
